# Tracking footprints of artificial and natural selection signatures in breeding and non-breeding cats

**DOI:** 10.1038/s41598-022-22155-7

**Published:** 2022-10-27

**Authors:** Xuying Zhang, Kokila Jamwal, Ottmar Distl

**Affiliations:** Institute for Animal Breeding and Genetics, University of Veterinary Medicine Hannover, Hannover, Germany

**Keywords:** Genetics, Diseases

## Abstract

Stray non-breeding cats (stray) represent the largest heterogeneous cat population subject to natural selection, while populations of the Siamese (SIAM) and Oriental Shorthair (OSH) breeds developed through intensive artificial selection for aesthetic traits. Runs of homozygosity (ROH) and demographic measures are useful tools to discover chromosomal regions of recent selection and to characterize genetic diversity in domestic cat populations. To achieve this, we genotyped 150 stray and 26 household non-breeding cats (household) on the Illumina feline 63 K SNP BeadChip and compared them to SIAM and OSH. The 50% decay value of squared correlation coefficients (*r*^*2*^) in stray (0.23), household (0.25), OSH (0.24) and SIAM (0.25) corresponded to a mean marker distance of 1.12 Kb, 4.55 Kb, 62.50 Kb and 175.07 Kb, respectively. The effective population size (*N*_*e*_) decreased in the current generation to 55 in stray, 11 in household, 9 in OSH and 7 in SIAM. In the recent generation, the increase in inbreeding per generation (*ΔF*) reached its maximum values of 0.0090, 0.0443, 0.0561 and 0.0710 in stray, household, OSH and SIAM, respectively. The genomic inbreeding coefficient (*F*_*ROH*_) based on ROH was calculated for three length categories. The *F*_*ROH*_ was between 0.014 (*F*_*ROH60*_) and 0.020 (*F*_*ROH5*_) for stray, between 0.018 (*F*_*ROH60*_) and 0.024 (*F*_*ROH5*_) for household, between 0.048 (*F*_*ROH60*_) and 0.069 (*F*_*ROH5*_) for OSH and between 0.053 (*F*_*ROH60*_) and 0.073 (*F*_*ROH5*_) for SIAM. We identified nine unique selective regions for stray through genome-wide analyses for regions with reduced heterozygosity based on *F*_*ST*_ statistics. Genes in these regions have previously been associated with reproduction (*BUB1B*), motor/neurological behavior (*GPHN, GABRB3*), cold-induced thermogenesis (*DIO2*, *TSHR*), immune system development (*TSHR*), viral carcinogenesis (*GTF2A1*), host immune response against bacteria, viruses, chemoattractant and cancer cells (*PLCB2*, *BAHD1*, *TIGAR*), and lifespan and aging (*BUB1B*, *FGF23*). In addition, we identified twelve unique selective regions for OSH containing candidate genes for a wide range of coat colors and patterns (*ADAMTS20*, *KITLG, TYR*, *TYRO3*—a *MITF* regulator, *GPNMB*, *FGF7*, *RAB38*) as well as congenital heart defects (*PDE4D*, *PKP2*) and gastrointestinal disorders (*NLGN1*, *ALDH1B1*). Genes in stray that represent unique selective events indicate, at least in part, natural selection for environmental adaptation and resistance to infectious disease, and should be the subject of future research. Stray cats represent an important genetic resource and have the potential to become a research model for disease resistance and longevity, which is why we recommend preserving semen before neutering.

## Introduction

Early evidence for human-cat coexistence comes from a wildcat interred near a human in Cyprus approximately 9500 years ago^1^. Data from early Chinese agricultural settlements dated between 5560–5280 B.P. suggest mutualistic commensal relationships between cats and people^2^. The domestic cat (*Felis silvestris catus*) is one of the most popular pet species. The *Felidae* family includes 40–71 recognized domestic cat breeds distributed throughout the world^3–5^, with considerable variation in coat, behavioral, and morphological phenotypes^6^. The spectacular phenotypic diversity of most domestic cat breeds is thought to have arisen recently, within the past 150 years, largely through intensive artificial selection for aesthetic rather than functional traits^7^.

The Siamese cat (SIAM), which originated in the Kingdom of Siam (now Thailand), is one of the first distinctly recognized breeds. The breed was already known in the fourteenth century and became one of the most popular breeds in Europe and North America in the nineteenth century^8^. Starting in the mid-twentieth century, modern SIAM became established, characterized by blue almond-shaped eyes, a very slender body, and wedge-shaped head. SIAM are usually very affectionate and intelligent and are known to be natural conversationalists^9^. Developed from SIAM, Oriental Shorthair (OSH) was first recognized as a distinct breed by the CFA (USA) in 1977^9^. The OSH has a similar body shape to the modern SIAM but comes in various solid colors and patterns such as smoke, shaded, particolor/tortoiseshell, tabby and bicolor^10^. A study showed that SIAM living on European mainland had mean inbreeding coefficients per birth year ranging from 0.03 to 0.12 in data comprising animals born between 1920 and 2000. Individual inbreeding coefficients were calculated from diagonal elements of the kinship matrix. Average inbreeding coefficients in 1998–1999 were 0.12 and 0.10 in SIAM and OSH, respectively^11^. Based on Swedish insurance data, SIAM and SIAM-derived breeds had a higher mortality rate compared to other breeds with a median lifespan of 10–14.2 years in SIAM^12^. OSH and SIAM have similar health problems: neurodegenerative disease, amyloidosis, asthma/bronchial disease, congenital heart defects, crossed eyes, gastrointestinal conditions, hyperesthesia syndrome, lymphoma, nystagmus and progressive retinal atrophy^9^. The G302R mutation in *TYR* causes the ‘pointed’ coat color phenotype in SIAM (100% alternate allele frequency, *n* = 9)^13,14^. The causative mutation of retinal degenerative disease in *CEP290* had a frequency of ∼33% in North American and European SIAM populations^15^. Neurological disease phenotypes in SIAM arose from polymorphisms within *GLB1* (GM1 gangliosidosis)^16,17^ and *ABCB1* (macrocyclic lactone–induced neurologic toxicosis, 1.85% risk allele frequency)^18^. Metabolic disorder phenotypes in SIAM are associated with genetic variants in *ARSB* (mucopolysaccharidosis type VI, 11.4% risk allele frequency)^19^ and *SLC7A9* (cystinuria)^20^.

Measures of demographic indicators and genomic architecture of cat breeds have been performed based on pedigree^11^, SNP-array^21,22^ or whole genome sequencing (WGS)^23^ data. A summary of previous studies is given in Supplementary Table S1. Illumina Infinium iSelect 63 K DNA feline array demonstrated its usefulness in performing population-based investigations^24^. To date, ROH analysis in cats has identified selection signatures that shaped the Persian breed^22^, and common ROH islands for multiple populations^25^. An ROH island on chromosome B3 at 25–29 Mb was identified in SIAM, OSH, and other nine domestic cat populations^25^. The candidate gene *ARID3B* in this region, known to impact neural crest cell survival, was linked to a domestication syndrome hypothesis^23,26^. The measures *F*_*IS*_, *F*_*ST*_, and *F*_*IT*_ are collectively referred to as *F-statistics* and are derived from the inbreeding coefficient. They are related to the amount of heterozygosity at various levels of population structure. *F*_*IS*_ and the summary statistic d_i_ based on pairwise *F*_*ST*_ have been used to determine selection signatures in more than 26 domestic cat breeds^5,21,23^. A shared candidate region on chromosome D3 (1.25–2.25 Mb) has been identified in SIAM, OSH and Peterbald breeds, which share a slender body shapes as a common trait^5^.

Stray and household cats are collectively referred to as non-breed, random bred or incidentally bred domestic cats. These non-breed cats are usually not artificially selected or bred, but are subject to natural selection (Supplementary Table S2). Using 15 short tandem repeat (STR) markers, average observed heterozygosity (*Ho* = 61% ± 5%) and the degree of inbreeding level (average fixation Index *F*_*IS*_ = 0.14 ± 0.05) had been studied in Australian household-stray cats^27^. However, a refined assessment based on genome-wide BeadChip array data had not been performed in stray cats. According to cat statistics (http://carocat.eu/), there are more than 480 million stray cats worldwide, accounting for 80% of the total number of cats. Stray cats have an abundant variation in phenotypes in terms of coat type and color, eye color, eye shape, eye pupil, ears, tail, shade, and body^28^. Stray cats have a high reproductive potential, and tend to transmitting various infectious and parasitic diseases, as well as viruses^29–31^. Presumably, stray cats could represent an important genetic resource of various morphological, physiological and adaptability traits that would facilitate animal breeding in the future under challenging conditions. For example, the rexoid mutation, one of the most recent mutations, arose from a random bred population in the United Kingdom to develop the Devon Rex breed^32,33^. Characterizing the genomic diversity and possible natural selection signatures of stray cats is essential for optimal genetic resources management.

In the present study, we analyze genotypic variants based on the feline 63 K SNP BeadChip array in stray, household, OSH and SIAM. Stray cats include randomly selected individuals from eight different regions in Germany. The objective of this study was to assess genomic diversity and detect signatures of natural and artificial selection in the non-breed and breed populations. Moreover, we estimated genomic inbreeding from ROHs *F*_*ROH*_ and fixation indices *F*_*IS*_ in the populations.

## Results

### Measures of diversity and population stratification

**Figure 1:**
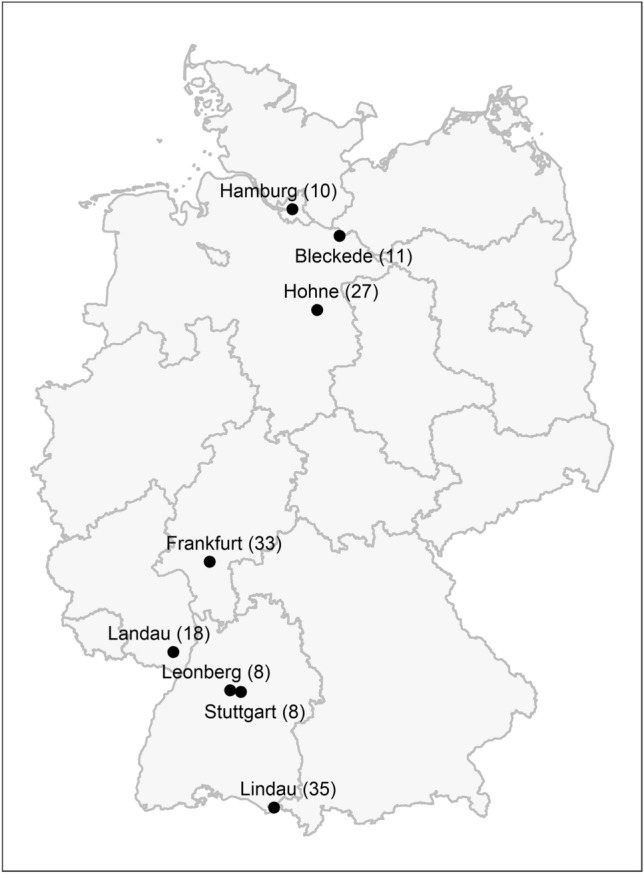
Sampling locations of stray cats in Germany. (N) indicates the number of stray cats genotyped at each sampling location. The map was created using raster of the R package, version 3.5–29 (https://CRAN.R-project.org/package=raster).

The feline 63 K SNP array contains 62,897 SNPs. After data filtering and quality control, the final dataset contained 53,581 autosomal SNPs with a genotyping rate > 0.95 in 246 cats. The SNP distribution across the autosomal cat genome in 1 Mb window sizes can be considered homogeneous, with a SNP density of 0.02241/Kb. The dataset included 150 stray from 8 residential regions (Fig. 1), defined as 8 stray subpopulations, 26 household, 49 OSH and 21 SIAM (Table 1). Stray and household were randomly selected individuals and breed cats were unrelated according to their pedigree records. The minor allele frequency (MAF) was 0.21 in stray and household, and 0.15 in OSH and SIAM. In stray and household, the observed heterozygosity (*H*_*O*_) was greater than the expected heterozygosity (*H*_*E*_). In OSH and SIAM, however, *H*_*O*_ is smaller than *H*_*E*_. *H*_*O*_ in stray, household, OSH and SIAM was 0.714, 0.704, 0.722 and 0.672, respectively. *H*_*O*_ in the stray subpopulations ranged from 0.701 for Stuttgart-stray to 0.726 for Leonberg-stray. The percentage of monomorphic SNPs were lower in stray (3.73%) and household (9.62%) compared to that in OSH (22.63%) and SIAM (35.43%).

**Table 1:** Population summaries of percentage of monomorphic SNPs (% Mono), minor allele frequency (MAF), observed heterozygosity (*H*_*O*_), expected heterozygosity (*H*_*E*_), and fixation index (*F*_*IS*_), as well as linkage disequilibrium (LD) estimates of cat populations.

Population	Resident-ial region	*n*	% Mono	MAF	*H*_*O*_	*H*_*E*_	*F*_*IS*_	LD at 50% Decay	
								*r*^*2*^	Distance (Kb)
Stray		150	3.73	0.213	0.714	0.698	0.052	0.23	1.12
	Bleckede	11			0.711	0.698	0.045		
	Frankfurt	33			0.718	0.698	0.067		
	Hamburg	10			0.721	0.698	0.076		
	Hohne	27			0.709	0.698	0.037		
	Landau	18			0.714	0.698	0.054		
	Leonberg	8			0.726	0.698	0.092		
	Lindau	35			0.711	0.698	0.045		
	Stuttgart	8			0.701	0.698	0.012		
Household		26	9.62	0.206	0.704	0.689	0.047	0.25	4.55
Oriental shorthair		49	22.63	0.153	0.722	0.730	− 0.031	0.24	62.50
Siamese		21	35.43	0.146	0.672	0.693	− 0.071	0.25	175.07
Total cats		246			0.711	0.703	0.025		

**Figure 2:**
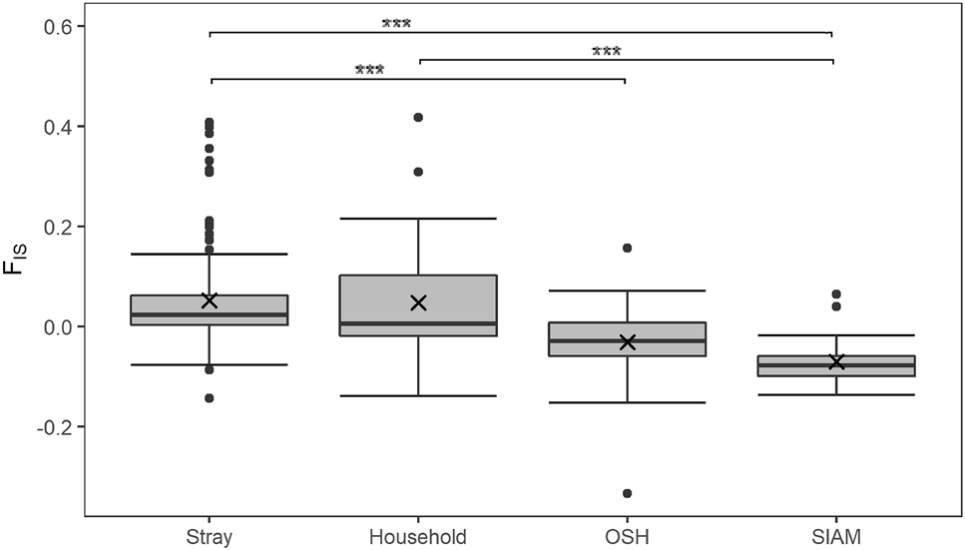
Distribution of the fixation index F_IS_. Outliers are shown as dots. Median and mean values are indicated with a thick horizontal line and a cross. ***p < 0.0001.

A positive mean of fixation index (*F*_*IS*_) was calculated in stray (0.052 ± 0.094) and household (0.047 ± 0.12), while a negative mean was obtained for OSH (− 0.031 ± 0.072) and SIAM (− 0.071 ± 0.050). *F*_*IS*_ values differed significantly different between non-breed and breed populations (*p* < *0.0001*) (Fig. 2). However, the *F*_*IS*_ of different stray subpopulations did not differ significantly (Supplementary Table S3) and did not show clusters based on their geographic distribution (Supplementary Fig. S1).

**Figure 3:**
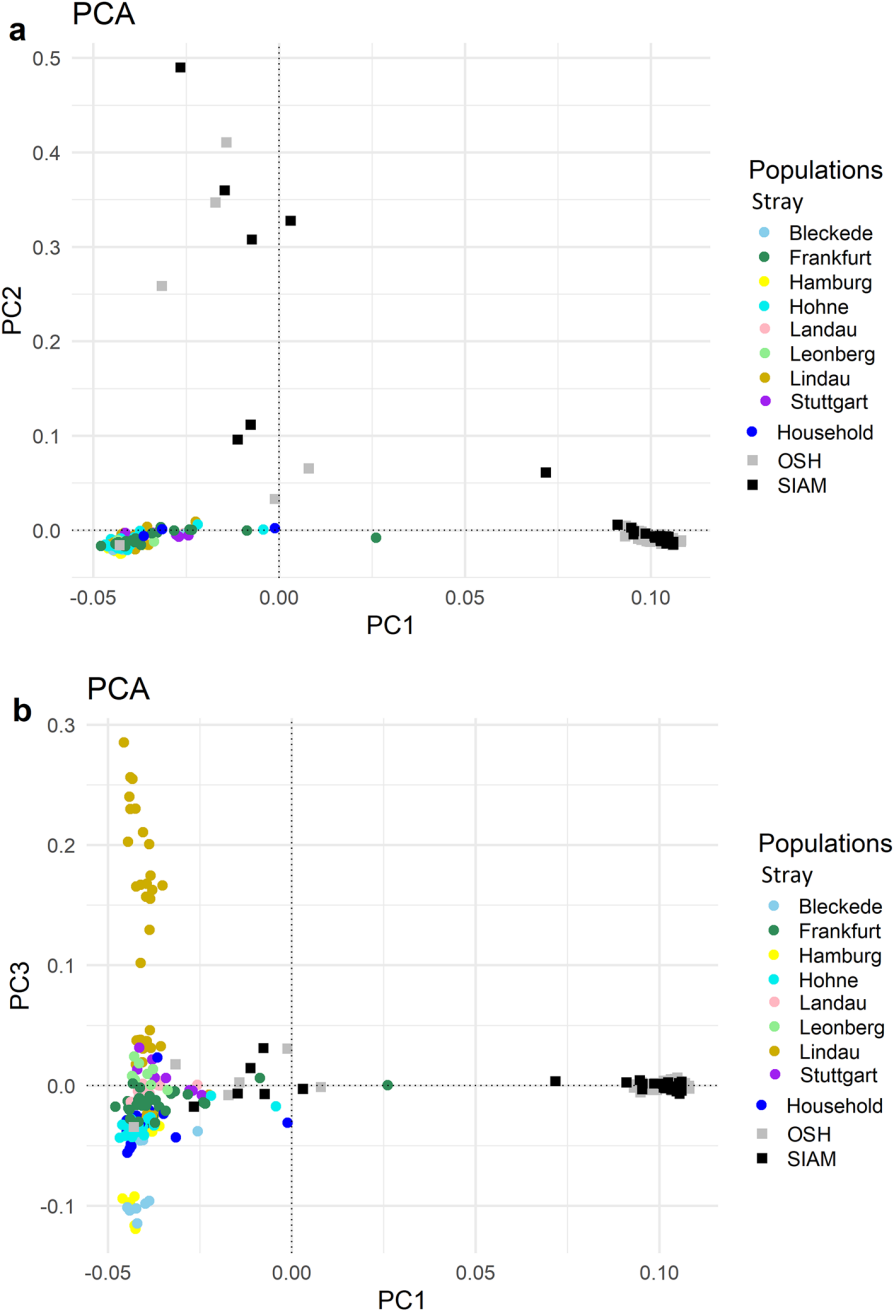
Principal component analysis of cat populations. Distances between cat populations are plotted in two dimensions for PC1 vs. PC2 in (**a**), PC1 vs. PC3 in (**b**). Non-breeding cats are highlighted by colored dots, each colored dot corresponding to a household population or a subpopulation of stray living in different regions of Germany (Bleckede, Frankfurt, Hamburg, Hohne, Landau, Leonberg, Lindau and Stuttgart). The black and grey squares represent the SIAM and OSH breeds.

The principal components analysis (PCA) plots in Fig. 3a,b showed a separation between breed cats (OSH, SIAM) and non-breed cats (stray, household), for the first three PCs. A distribution from left to right was observed, with stray and domestic cats on the left side of the diagram, while OSH and SIAM cats were distributed from the centre to the right. PCs of OSH and SIAM overlap almost completely. Means of PC1 and PC2 values of stray and household were not significantly different from each other, nor did they show clusters based on their geographical distribution (Supplementary Fig. S2). Nevertheless, stray from Frankfurt clustered in a wider range for PC1 and stray from Lindau for PC3. Stray from these areas may be less related with each other than stray from other areas (Fig. 3a,b). NetView analysis using the genomic relationship matrix just for stray did not converge and could not cluster all stray subpopulations as one community (Supplementary Fig. S3). With exception of the residential regions Hohne and Leonberg, separate subclusters were obtained for the other areas. More than 50% of the cats from Hamburg (90%), Bleckede (73%) and Lindau (63%) were outside the main cluster.

### Linkage disequilibrium

**Figure 4:**
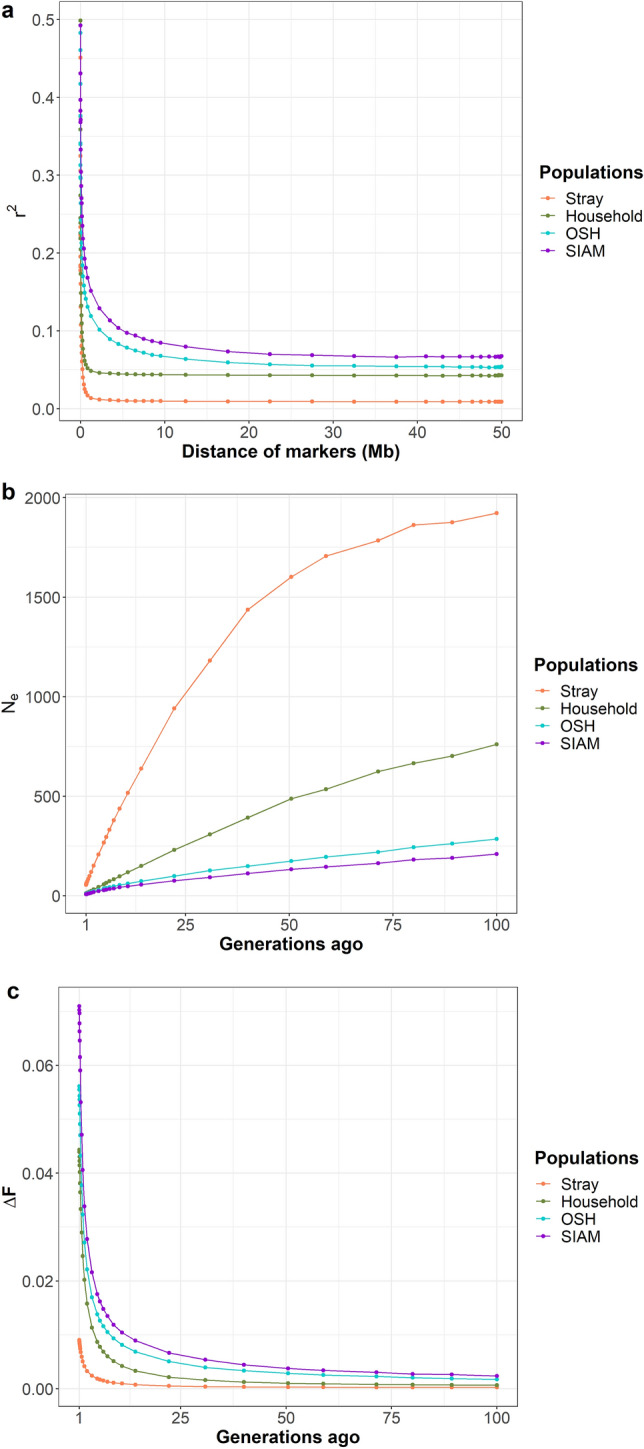
Analysis of linkage disequilibrium among SNP loci. (**a**) Decay of linkage disequilibrium (r^2^) between SNP pairs bridging an increasing distance in stray, household, OSH and SIAM, respectively. (**b**) Effective population size (Ne) of each population over the last 100 generations. The ancestral Ne was estimated from the mean r^2^ for the 18 cat autosomes. (**c**) Increase in inbreeding (ΔF) in each population in the last 100 generations.

The maximum mean *r*^*2*^ values in the stray, household, OSH and SIAM were 0.45, 0.50, 0.48 and 0.49, respectively (Fig. 4a). The 50% decay value of *r*^*2*^ in stray (0.23), household (0.25), OSH (0.24) and SIAM (0.25) corresponded to a mean marker distance of 1.12 Kb, 4.55 Kb, 62.50 Kb and 175.07 Kb, respectively (Table 1). Mean *r*^*2*^ values were reached for SNPs spaced 1.25 Mb apart, both for stray and household, with *r*^*2*^ values below 0.014 and0.049 for stray and household, respectively. Mean *r*^*2*^ values decreased to 0.060 for OSH and 0.074 for SIAM, both for SNPs spaced 17.48 Mb apart. In the last 100 generations, the effective population size (*N*_*e*_) decreased from 1923 to 55 in stray, from 760 to 11 in household, from 286 to 9 in OSH, and from 210 to 7 in SIAM, respectively (Fig. 4b). In the current generation, the increase in inbreeding per generation (*ΔF*) reached its maximum values of 0.0090, 0.0443, 0.0561 and 0.0710 in stray, household, OSH and SIAM, respectively (Fig. 4c).

### Runs of homozygosity and genomic inbreeding

#### Comparisons among the four populations

**Figure 5:**
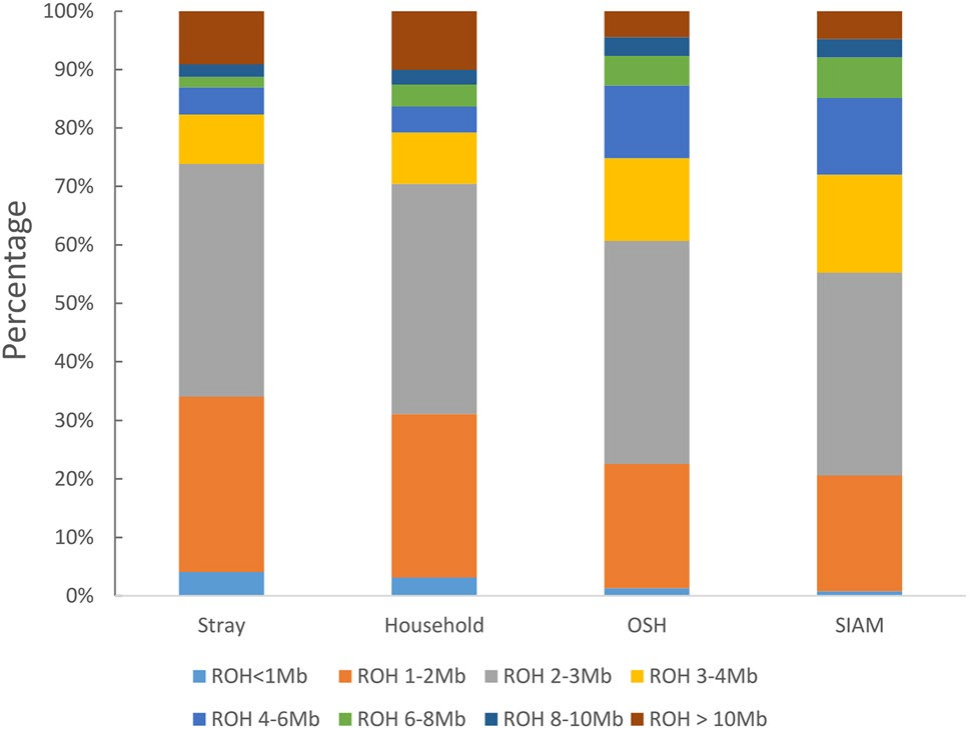
Percentage of ROH5 in eight different length ranges in stray, household, OSH and SIAM. The calculation was based on individual patterns of ROHs, i.e. the total number of ROHs for individuals in a population.

**Figure 6:**
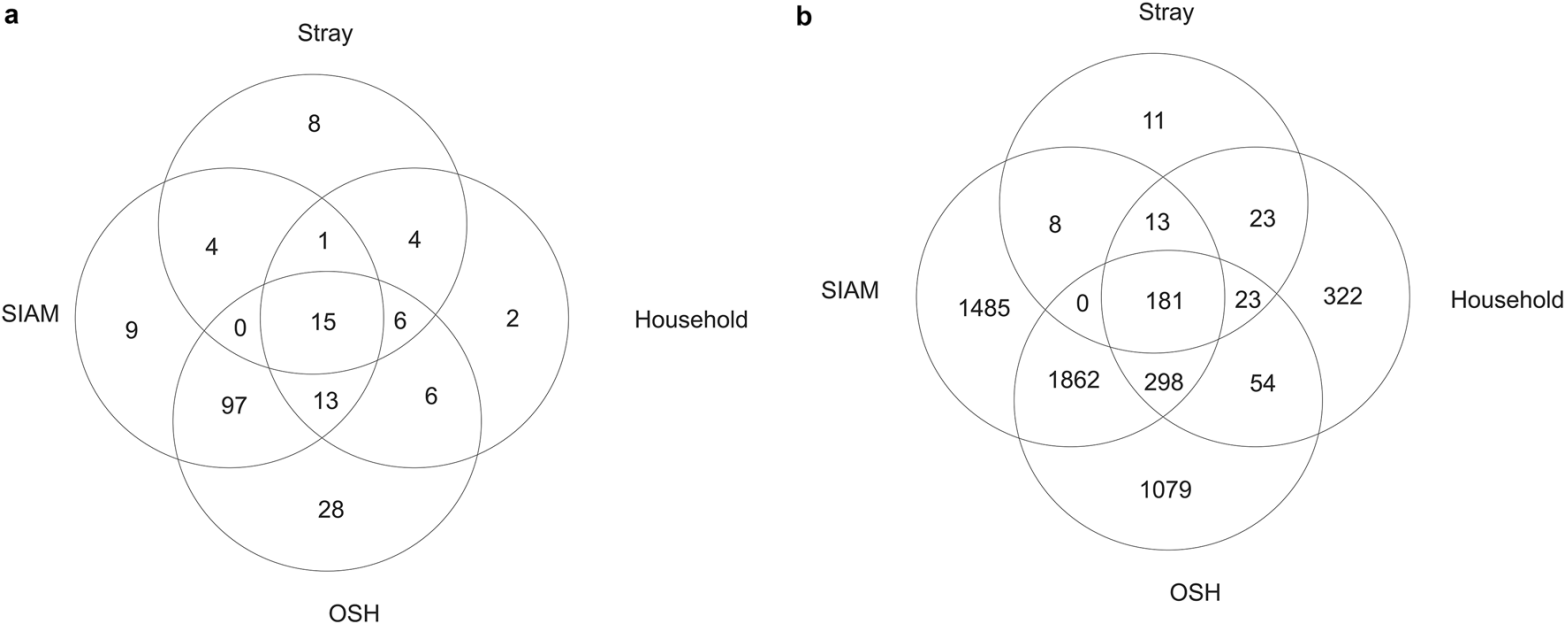
Comparison of the 10% consensus ROHs of stray, household, OSH and SIAM. Shown are the number of ROHs (**a**) and the number of genes in the ROHs (**b**).

Stretches of homozygosity were calculated based on SNP numbers of 5 (ROH5), 30 (ROH30) or 60 (ROH60) in the region. Based on individual patterns, we identified 712 (ROH60) to 1645 (ROH5) ROHs in stray (Table 2, Supplementary Table S4). ROH5 length in stray varied from 0.28 to 68.71 Mb with an average length of 4.25 Mb. In household individuals, 149 (ROH60) to 318 (ROH5) ROHs were identified with an average ROH5 length of 4.65 Mb (0.55–64.27 Mb). In OSH individuals, 1187 (ROH60) to 2136 (ROH5) ROHs were detected and the ROH5 had an average length of 3.69 Mb (0.22 Mb-33.11 Mb). In SIAM individuals, there were 551 (ROH60) to 922 (ROH5) ROHs and the ROH5 had an average length of 3.88 Mb (0.39–29.31 Mb). Percentages of ROHs in the different length categories were calculated based on individual patterns (Fig. 5, Supplementary Fig. S4). Stray and household had a larger number of shorter ROH with up to 3 Mb (74 and 70%) than OSH (61%) and SIAM (55%). We identified one household and a few stray individuals with more than five ROH > 10 Mb. These cats caused the larger proportion of ROH > 10 Mb in stray and household compared with OSH and SIAM. Individuals of stray, household, OSH or SIAM did not share a 100% consensus population-specific ROH (Table 3). Thirty-eight, 47, 165 and 139 ROHs reached a 10% consensus level in stray (Supplementary Table S5), in household (Supplementary Table S6), in OSH (Supplementary Table S7) and in SIAM, respectively (Supplementary Table S8). Comparisons among populations were made for 10% consensus ROHs from stray, household, OSH and SIAM, respectively. Of the 10% consensus ROHs, 15 from stray overlapped with household, SIAM and OSH as well as 26, 21 and 20 from stray overlapped with household, OSH and SIAM, respectively (Fig. 6a). Eight of the 10% consensus ROHs of stray could not be identified in other populations and 11 genes are located in these ROHs (Fig. 6b). In the 15 10% consensus ROHs of stray overlapping with those of the other three populations, 181 genes are located.

**Table 2:** Individual patterns of runs of homozygosity (ROHs) for the four cat populations. ROH5, ROH30 and ROH60 comprised windows of at least 5, 30 and 60 SNPs, respectively.

Population	ROH	Overall number of ROHs	Length of shortest ROH (Mb)	Mean length of ROH (Mb)	Length of longest ROH (Mb)	Cumulative length of all ROHs (Gb)	Mean *F*_*ROH*_ (range)
Stray, *n* = 150	ROH5	1645	0.28	4.25	68.71	6.99	0.0201 (0.000–0.113)
	ROH30	1564	1.23	4.43	68.72	6.93	0.0135 (0.000–0.102)
	ROH60	712	2.40	7.35	68.72	5.24	0.0135 (0.000–0.102)
Household, *n* = 26	ROH5	318	0.55	4.65	64.27	1.48	0.0245 (0.002–0.107)
	ROH30	306	1.21	4.80	64.27	1.47	0.0176 (0.00–0.101)
	ROH60	149	2.40	7.78	64.27	1.16	0.0176 (0.000–0.101)
OSH, *n* = 49	ROH5	2136	0.22	3.69	33.11	7.88	0.0692 (0.009–0.100)
	ROH30	2106	1.29	3.73	33.11	7.86	0.0475 (0.001–0.08)
	ROH60	1187	2.40	5.08	33.11	6.03	0.0475 (0.001–0.08)
SIAM, *n* = 21	ROH5	922	0.39	3.88	29.31	3.58	0.0734 (0.046–0.107)
	ROH30	914	1.20	3.91	29.31	3.57	0.0531 (0.025–0.091)
	ROH60	551	2.40	5.17	29.31	2.85	0.0531 (0.025–0.091)

For all three different classifications of ROHs, number and length of detected ROHs and the inbreeding coefficient *F*_*ROH*_ for each of the four populations are given. In total, the genotyped single nucleotide polymorphisms (SNPs) covered 2,322,306,946 bp of the autosomal cat genome.

**Table 3:** Overview on the number and length of the ROHs (ROH5) shared by at least 10, 20, 25, 30, 50, 75, 90, or 100% of the genotyped cats in OSH, SIAM, stray and household, respectively.

Degree of consensus	Stray (*n* = 150)			Household (*n* = 26)			OSH (*n* = 49)			SIAM (*n* = 21)		
	*n* of ROHs	Mean length (Mb)	Cumulative length (Mb)	*n* of ROHs	Mean length (Mb)	Cumulative length (Mb)	*n* of ROHs	Mean length (Mb)	Cumulative length (Mb)	*n* of ROHs	Mean length (Mb)	Cumulative length (Mb)
10%	38	2.11	80.23	47	3.65	171.53	165	4.05	668.39	139	5.42	752.77
20%	7	1.59	11.13	21	2.02	42.51	113	2.14	241.39	115	3.24	372.20
25%	8	0.30	2.41	13	1.14	14.87	78	1.98	154.34	86	3.00	258.26
30%	0	–	–	7	1.27	8.86	42	2.06	86.42	84	2.09	175.86
50%	0	–	–	0	–	–	12	1.30	15.58	24	2.09	50.20
75%	0	–	–	0	–	–	1	2.27	2.27	4	2.53	10.11
90%	0	–	–	0	–	–	0	–	–	2	1.17	2.35
100%	0	–	–	0	–	–	0	–	–	0	–	–

For all degrees of consensus, the number (*n*) and the mean length of ROHs found as well as the cumulative length of all ROHs are given.

**Figure 7:**
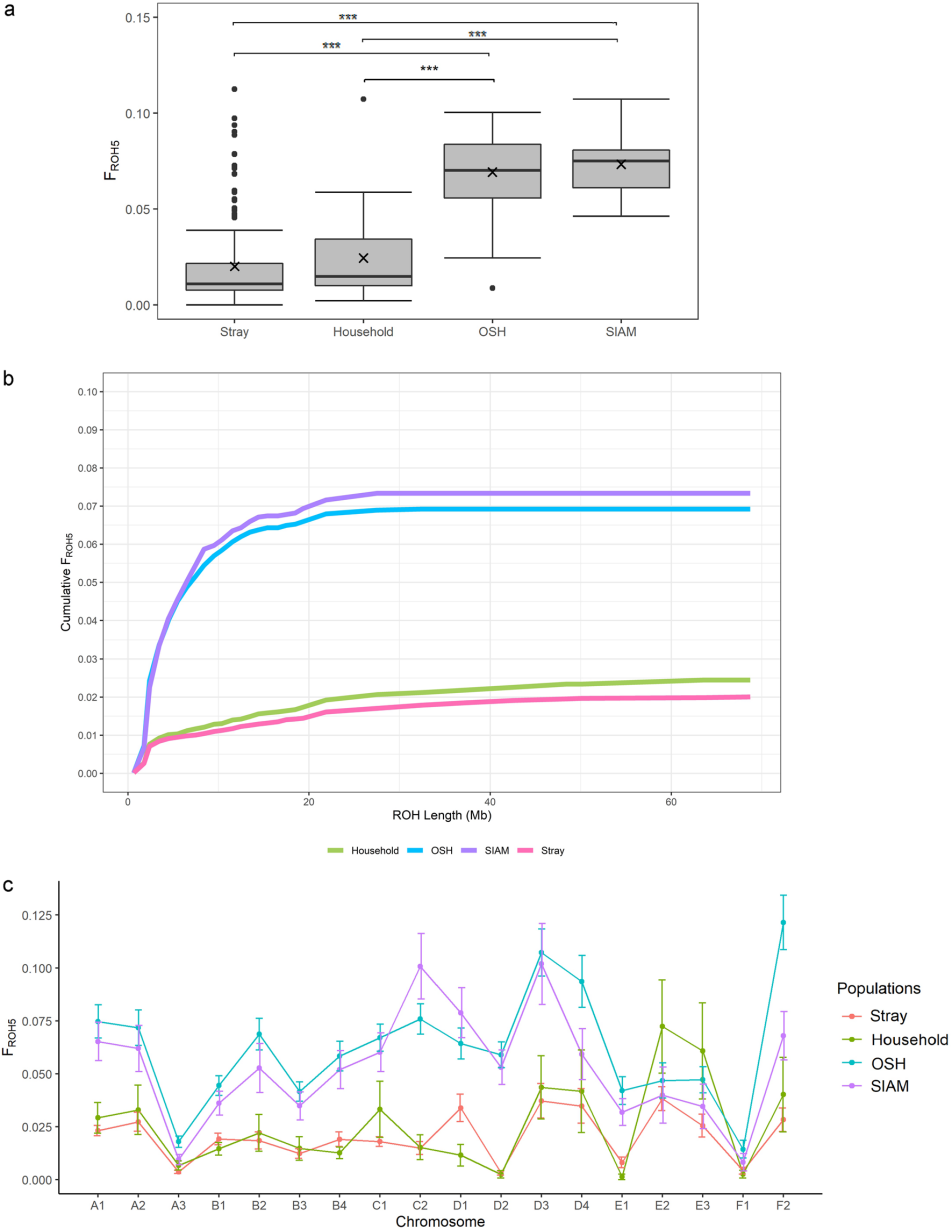
Genomic inbreeding analyses in the four genotyped cat populations. The ROHs detected over ROH5 were analysed. (**a**) Distribution of the genomic inbreeding coefficient FROH5. Outliers are shown as dots. Median and mean values are indicated with a thick horizontal line and a cross. ***p < 0.0001 (**b**) Cumulative genomic inbreeding coefficients for stray, household, OSH and SIAM by ROH lengths in windows of Mb. (**c**) Average genomic inbreeding for the 18 autosomes by stray, household, OSH and SIAM.

The mean inbreeding coefficients over ROH5 (*F*_*ROH5*_) were 0.020 ± 0.022, 0.024 ± 0.024, 0.069 ± 0.020 and 0.073 ± 0.015 for stray, household, OSH and SIAM, respectively. Corresponding median values of *F*_*ROH5*_ were 0.011, 0.015, 0.070 and 0.075. Means and medians of *F*_*ROH5*_ were significantly (*p* < *0.0001*) lower in stray and household than in OSH and SIAM (Fig. 7a). Differences in genomic inbreeding coefficients *F*_*ROH5*_ were not significantly different among residential regions. The highest value was seen for stray from Leonberg (0.031) and the lowest for stray from Hohne (0.015). Genomic inbreeding coefficients per population across the different ROH lengths showed that all four cat populations had a very similar in distribution of ROH up to genomic inbreeding coefficients of 0.01 (Fig. 7b). This proportion of genomic inbreeding can be attributed to shorter ROH segments (< 7.5 Mb). In stray, further increase of genomic inbreeding through longer ROH segments decreased more and more with longer ROH segments. However, in OSH and SIAM, increase of genomic inbreeding continued with longer ROH segments up to their final values of genomic inbreeding coefficients. Mean *F*_*ROH5*_ by chromosomes ranged from 0.31% (D2) to 3.82% (E2) in stray and from 0.12% (E1) to 7.24% (E2) in household (Fig. 7c). On 16 chromosomes except E2 and E3, OSH and SIAM had higher mean *F*_*ROH5*_ than stray and household. Mean *F*_*ROH5*_ in OSH and SIAM ranged from 1.43% (F1) to 12.15% (F2) and from 0.82% (F1) to 10.20% (D3), respectively. Pearson correlation coefficients between *F*_*IS*_ and *F*_*ROH5*_ based on different ROH lengths for the four populations ranged from 0.755 to 0.999 (Table 4).

**Table 4:** Pearson correlation coefficients between genomic inbreeding coefficient *F*_*ROH5*_ based on different ROH lengths (all, > 1 Mb and > 4 Mb) and fixation index *F*_*IS*_ by the four populations.

	*F*_*IS*_	*F*_*ROH5*_all_	*F*_*ROH5*>1 Mb_	*F*_*ROH5*>4 Mb_
**Inbreeding measures in stray**				
*F*_*IS*_	1			
*F*_*ROH5*_all_	0.89194	1		
*F*_*ROH5*>1 Mb_	0.89190	0.99996	1	
*F*_*ROH5*>4 Mb_	0.86847	0.99433	0.99429	1
**Inbreeding measures in household**				
*F*_*IS*_	1			
*F*_*ROH5*_all_	0.84439	1		
*F*_*ROH5*>1 Mb_	0.84463	0.99996	1	
*F*_*ROH5*>4 Mb_	0.83533	0.99319	0.99336	1
**Inbreeding measures in OSH**				
*F*_*IS*_	1			
*F*_*ROH5*_all_	0.83905	1		
*F*_*ROH5*>1 Mb_	0.83927	0.99996	1	
*F*_*ROH5*>4 Mb_	0.75491	0.96618	0.96646	1
**Inbreeding measures in SIAM**				
*F*_*IS*_	1			
*F*_*ROH5*_all_	0.77505	1		
*F*_*ROH5*>1 Mb_	0.77415	0.99997	1	
*F*_*ROH5*>4 Mb_	0.76893	0.95185	0.95142	1

#### Comparison among the eight stray subpopulations

**Figure 8:**
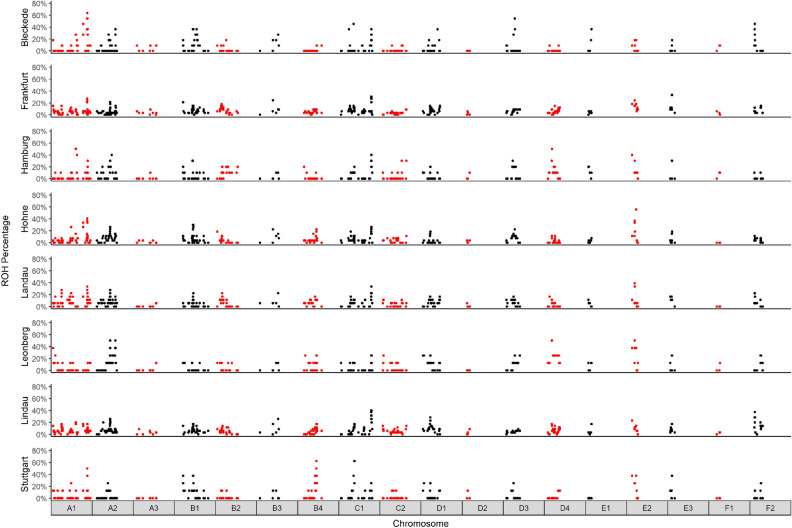
Distribution of partial consensus ROHs in stray. Shown are the distributions of ROH percentages in eight stray subpopulations across all autosomes. The alternating red and black ROH percentages are from neighboring chromosomes.

In stray, we detected 391 partial consensus ROHs in total (Fig. 8, Supplementary Table S9). Twenty-two of the 391 stray partial consensus ROHs were shared by stray cats from eight different residential regions, with a low consensus level between 0.07 and 0.29 (Table 5). Thirty-nine stray partial consensus ROHs were detected in stray cats from each two different residential regions (Table 6). Leonberg and Stuttgart are cities, geographically close to each other, but stray from these regions did even not share a partial consensus ROH.

**Table 5:** Partial consensus ROHs shared by stray cats from eight different residential regions.

Chr	Start (bp)	End (bp)	*No* (percentage) of animals	*No* (percentage) of stray cats from different regions containing ROHs							
				Bleckede	Frankfurt	Hamburg	Hohne	Landau	Leonberg	Lindau	Stuttgart
A2	93,141,879	93,673,705	13 (0.09)	3 (0.27)	1 (0.03)	2 (0.20)	2 (0.07)	1 (0.06)	1 (0.13)	2 (0.06)	1 (0.13)
A2	104,152,061	104,178,994	36 (0.24)	2 (0.18)	7 (0.21)	3 (0.30)	7 (0.26)	4 (0.22)	3 (0.38)	9 (0.26)	1 (0.13)
A2	104,251,009	104,698,329	36 (0.24)	2 (0.18)	7 (0.21)	3 (0.30)	6 (0.22)	4 (0.22)	4 (0.50)	9 (0.26)	1 (0.13)
A2	103,910,419	103,910,419	35 (0.23)	2 (0.18)	6 (0.18)	3 (0.30)	7 (0.26)	5 (0.28)	3 (0.38)	8 (0.23)	1 (0.13)
A2	102,886,573	102,985,616	22 (0.15)	2 (0.18)	2 (0.06)	1 (0.10)	5 (0.19)	3 (0.17)	2 (0.25)	6 (0.17)	1 (0.13)
A2	102,746,003	102,872,209	21 (0.14)	2 (0.18)	3 (0.09)	1 (0.10)	4 (0.15)	3 (0.17)	1 (0.13)	6 (0.17)	1 (0.13)
A2	102,217,045	102,312,702	18 (0.12)	2 (0.18)	3 (0.09)	1 (0.10)	3 (0.11)	2 (0.11)	1 (0.13)	5 (0.14)	1 (0.13)
B1	44,685,353	44,783,774	22 (0.15)	2 (0.18)	7 (0.21)	2 (0.20)	2 (0.07)	1 (0.06)	1 (0.13)	5 (0.14)	2 (0.25)
B1	44,983,128	45,454,625	22 (0.15)	1 (0.09)	7 (0.21)	1 (0.10)	3 (0.11)	1 (0.06)	1 (0.13)	5 (0.14)	3 (0.38)
B3	114,572,955	116,057,844	11 (0.07)	2 (0.18)	1 (0.03)	1 (0.10)	3 (0.11)	1 (0.06)	1 (0.13)	1 (0.03)	1 (0.13)
C1	198,076,022	198,204,303	43 (0.29)	3 (0.27)	9 (0.27)	2 (0.20)	6 (0.22)	6 (0.33)	2 (0.25)	14 (0.40)	1 (0.13)
C1	197,828,378	197,972,131	42 (0.28)	2 (0.18)	10 (0.30)	2 (0.20)	6 (0.22)	6 (0.33)	1 (0.13)	14 (0.40)	1 (0.13)
C1	197,648,200	197,686,731	41 (0.27)	2 (0.18)	10 (0.30)	3 (0.30)	6 (0.22)	4 (0.22)	1 (0.13)	14 (0.40)	1 (0.13)
D3	54,151,710	54,325,768	19 (0.13)	3 (0.27)	3 (0.09)	2 (0.20)	4 (0.15)	3 (0.17)	1 (0.13)	1 (0.03)	2 (0.25)
E2	40,638,837	40,795,837	38 (0.25)	2 (0.18)	8 (0.24)	3 (0.30)	10 (0.37)	6 (0.33)	4 (0.50)	3 (0.09)	2 (0.25)
E2	40,927,526	40,958,612	38 (0.25)	2 (0.18)	8 (0.24)	3 (0.30)	10 (0.37)	7 (0.39)	3 (0.38)	3 (0.09)	2 (0.25)
E2	41,133,252	41,248,049	38 (0.25)	2 (0.18)	8 (0.24)	3 (0.30)	9 (0.33)	7 (0.39)	3 (0.38)	4 (0.11)	2 (0.25)
E2	49,630,467	49,847,368	36 (0.24)	2 (0.18)	6 (0.18)	1 (0.10)	15 (0.56)	1 (0.06)	3 (0.38)	5 (0.14)	3 (0.38)
E2	23,472,166	23,866,218	30 (0.20)	1 (0.09)	6 (0.18)	4 (0.40)	3 (0.11)	2 (0.11)	3 (0.38)	8 (0.23)	3 (0.38)
E2	42,249,690	42,335,360	27 (0.18)	1 (0.09)	5 (0.15)	1 (0.10)	5 (0.19)	6 (0.33)	3 (0.38)	4 (0.11)	2 (0.25)
E3	16,168,413	16,599,808	34 (0.23)	2 (0.18)	11 (0.33)	3 (0.30)	5 (0.19)	2 (0.11)	2 (0.25)	6 (0.17)	3 (0.38)
E3	15,871,626	15,871,626	32 (0.21)	1 (0.09)	11 (0.33)	3 (0.30)	4 (0.15)	2 (0.11)	2 (0.25)	6 (0.17)	3 (0.38)

Given are the chromosomal location, total number (and percentage) of stray cats containing ROHs, and the distribution (number and percentage) of animals among the different regions.

**Table 6:** Overview on the number of partial consensus ROHs shared by stray cats from two different residential regions.

Residential region	Bleckede	Frankfurt	Hamburg	Hohne	Landau	Leonberg	Lindau
Frankfurt	2						
Hamburg	0	3					
Hohne	0	3	0				
Landau	0	3	1	1			
Leonberg	0	1	1	1	0		
Lindau	1	11	1	5	3	1	
Stuttgart	0	1	0	0	0	0	0

The *F*_*ROH5*_ of stray from eight different residential regions did not differ significantly from each other (Supplementary Table S10) and did not show clusters based on their geographic distribution (Supplementary Fig. S5).

### Signatures of selection and functional annotation

#### Population unique selective events

**Figure 9:**
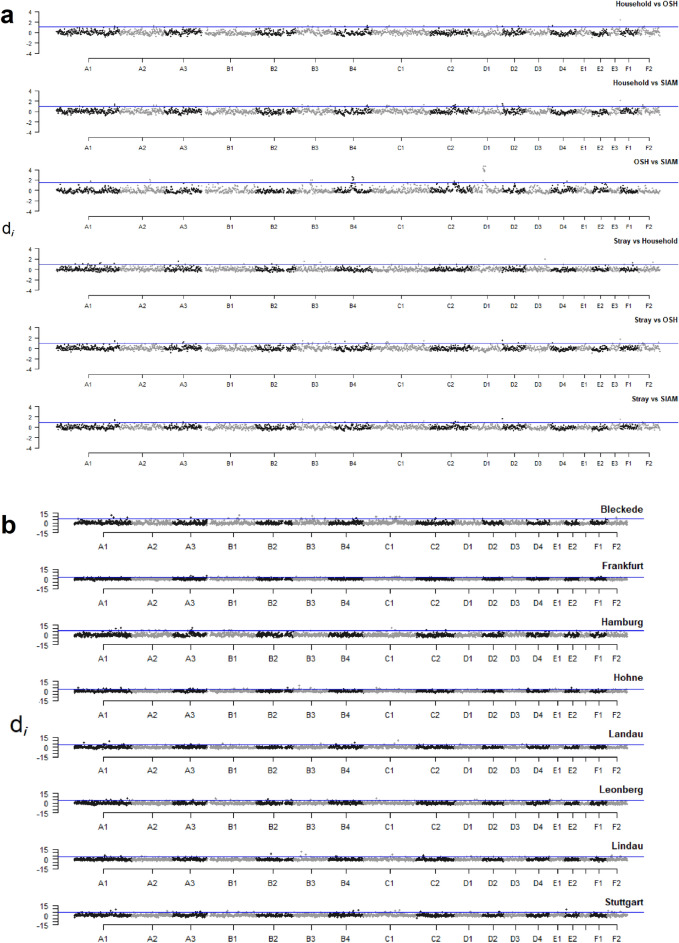
Distribution of the d_i_-summary statistics by cat populations and stray subpopulations. (**a**) The distributions of d_i_-summary statistics with stray compared to household, OSH and SIAM, respectively; household compared to stray, OSH and SIAM, respectively; OSH compared to stray, household, OSH and SIAM, are shown for each 1 Mb interval across all autosomes. (**b**) The distributions of d_i_-summary statistics with for each of the eight stray subpopulations compared to the other 7 stray subpopulations. Alternating grey and black indicate values in d_i_-summary statistics from neighboring chromosomes. The blue line indicates the 99th percentile for the populations.

The genome-wide distribution of locus specific divergence (*d*_*i*_) to evaluate population structure is shown in Fig. 9a. An incidence-plot of the SNPs per chromosome is given in Supplementary Fig. S6. In total, there were 137 1-Mb windows as outliers falling within the 99th percentile of the empirical distribution of *d*_*i*_. We defined scattering unique selective events in stray as the same outliers that appeared in *d*_*i, stray vs OSH*_, *d*_*i, stray vs SIAM*_, and *d*_*i, stray vs household*_, *but neither in d*_*i, household vs OSH*_ nor in *d*_*i, household vs SIAM*_. In this way, we excluded non-unique stray selective events that were also displayed as household selective events. Nine of the 137 significant 1-Mb windows on chromosomes A1, A3, B3, B4, C1 and F2 were identified as unique selective stray candidate regions (Table 7). Among them, regions on A1 at 221,005,995–221,993,341 bp and on B3 at 127,047,728–127,966,476 bp overlapped with the 10% consensus ROH blocks identified in stray. The region on chromosome B3 at 115,036,645–115,982,934 bp did not reach the 10% consensus level (7%), but overlapped with a partial consensus ROH shared by stray from eight different residential regions. There were 60 genes located in the 9 stray unique candidate selective regions. Ten genes of these play important roles in regulating lifespan, aging, reproduction, immune system development, immune response, viral carcinogenesis, cold-induced thermogenesis and motor/neurological behavior. Twenty- three 1-Mb significant windows on chromosomes A1, A2, B3, B4, C1, C2, D1 and D4, outliers in *d*_*i, OSH vs SIAM*_, were determined to be unique OSH candidates for selective events (Table 8). Two regions on C2 at 92,020,185–92,988,315 bp and 94,028,432–94,990,150 bp overlapped with 30% consensus ROH blocks, and two regions on A1 at 130,038,136–130,974,435 bp and on D4 at 61,062,534–61,997,601 bp overlapped with 10% consensus ROH blocks identified in OSH. Of the 95 genes located in the 12 OSH-unique selective candidate regions, 11 genes were associated with pigmentation, congenital heart defects and gastrointestinal disorders.

**Table 7:** Unique selective events identified by comparing of *d*_*i*_-summary statistics of stray with household, OSH and SIAM.

Chr	Start (bp)	End (bp)	Length(bp)	Stray subpop. specific	Overlapped with partial consensus ROHs in stray		*No* SNPs	*No* genes	Functions of those genes
					Consensus degree	*No of* living regions			
A1	221,005,995	221,993,341	987,346	Yes (BL, LE)	10%	7	27	0	
A3	71,015,071	71,946,584	931,513	No		–	11	1	
B3	25,010,984	25,942,926	931,942	No		–	23	2	Receptor for inhibitory neurotransmitter (*GABRB3*)^42^
B3	64,044,736	64,994,760	950,024	No		–	20	26	Regulation of normal aging (*BUB1B*)^51^, reproduction (*BUB1B*)^52^, immune response regulation (*PLCB2, BAHD1*)^48,49^
B3	115,036,645	115,982,934	946,289	No		8	25	16	Behavior/neurological (*GPHN*)^41^
B3	127,047,728	127,966,476	918,748	No	10%	7	26	4	T-cell development (*TSHR*)^47^, viral *carcinogenesis (GTF2A1)*^45,46^, cold-induced thermogenesis (*DIO2, TSHR*)^43,44^
B4	39,070,713	39,992,682	921,969	No		–	13	8	Apoptosis and autophagy (*TIGAR*)^50^, short lifespan, premature aging (*FGF23*)^53^
C1	60,022,511	60,999,850	977,339	No		4	27	1	
F2	81,001,554	81,998,016	996,462	No		–	16	2	

For each of the selective events, the chromosomal location, the estimated region length, the number of SNPs and the number of genes located in the respective region, as well as the functional annotation of these genes are given. For selective events that are specific to a subpopulation of stray (Stray subpop, specific) and overlap with a partial consensus ROH for stray, the degree of consensus level (%) and the number of residential regions from which stray cats were collected are given.

*BL* Bleckede, *LE* Leonberg.

**Table 8:** Unique selective events identified by comparing OSH with SIAM using *d*_*i*_-summary statistics.

Chr	Start (bp)	End (bp)	Length (bp)	No SNPs	No genes	Overlapping with partial consensus ROHs in OSH (consensus degree)	Functions of those genes
A1	130,038,136	130,974,435	936,299	18	4	10%	Cardioembolic stroke (*PDE4D*)^61^
A2	116,042,149	117,985,555	1,943,406	43	13		Melanin biosynthesis, development of retinal pigment epithelium and iris (*GPNMB*)^58^
B3	56,011,360	56,890,439	879,079	13	7		Skin hypopigmentation (*FGF7*)^59^
B3	63,015,020	63,950,955	935,935	14	18		*MITF* regulator in melanoma (*TYRO3*)^57^
B4	67,005,011	67,969,355	964,344	15	5		Arrhythmogenic right ventricular cardiomyopathy (*PKP2*)^62^
B4	70,056,541	72,975,983	2,919,442	52	11		Coat/hair pigmentation (*ADAMTS20*)^54^, hair color (*KITLG*)^55,56^
C1	134,037,968	134,975,802	937,834	19	2		
C2	92,020,185	92,988,315	968,130	23	2	30%	
C2	94,028,432	94,990,150	961,718	23	1	30%	Hirschsprung disease(*NLGN1*)^63^
C2	108,000,056	108,994,369	994,313	22	6		
D1	42,138,904	50,969,224	8,830,320	197	25		Ocular hypopigmentation (*RAB38*)^60^, coat color (*TYR*)^55^
D4	61,062,534	61,997,601	935,067	27	10	10%	Colorectal cancer (*ALDH1B1*)^64^

For each of the selective events, the chromosomal location, the estimated region length, the consensus degree (%), the number of SNPs and the number of genes located in the event, as well as the functional annotation of those genes are given.

Functional annotation of genes in cross-population-unique selective events was performed for stray and OSH, respectively, to gain insight into the biological processes influenced by the genes (Table 9, Supplementary Fig. S7, Supplementary Table S11). Analysis of the PANTHER gene list revealed a high percentage of genes involved in biological regulation, cellular processes, localization and metabolic processes, and a low percentage involved in developmental processes, locomotion, multicellular organismal processes, responses to stimulus and signaling in the selective events of stray and OSH. Reproduction and reproductive processes were only assigned to genes in the unique selective events of stray, biological adhesion and pigmentation only to the unique selective events of OSH.

**Table 9:** Functional annotations in unique selective events.

PANTHER gene ontology terms	Unique selective events in stray (%)	Unique selective events in OSH (%)
Biological adhesion	0.0	2.5
Biological regulation	17.6	13.2
Cellular process	28.6	30.8
Developmental process	3.3	5.7
Localization	11.0	11.3
Locomotion	2.2	1.3
Metabolic process	14.3	17.6
Multicellular organismal process	3.3	5.0
Pigmentation	0.0	0.6
Reproduction	1.1	0.0
Reproductive process	1.1	0.0
Response to stimulus	9.9	6.3
Signaling	7.7	5.7

PANTHER gene list analysis (http://www.pantherdb.org/) was performed for genes in unique selective regions in stray and OSH, respectively. The percent of gene hits against total number of process hits involved in specific biological processes are shown.

#### Stray subpopulations specific selective events

Stray subpopulation-specific selective events were identified by comparing one stray subpopulation with the other seven stray subpopulation *F*_*ST*_ results (*d*_*i, stray_sub*_) (Fig. 9b). We detected 23–24 subpopulation specific selective events for each of the eight stray subpopulations (Supplementary Table S12). Almost all selective events detected here were specific to only one subpopulation, ranging from 57% for Leonberg-stray subpopulation to 86% for Landau-stray subpopulation (Table 10). Whether two stray subpopulations share a selective event seemed to be independent of the geographic distance of their residential regions, e.g. there is only one shared selective event between the Leonberg- and Stuttgart-stray subpopulations, but five between the Leonberg- and Hohne-stray subpopulations. Of these unique selective stray events, only the region on chromosome A1 at 221,005,995–221,993,341 bp was specific to the stray subpopulations from Bleckede and Leonberg.

**Table 10:** Overview on the number of stray subpopulation-specific selective events.

Residential region	Bleckede	Frankfurt	Hamburg	Hohne	Landau	Leonberg	Lindau	Stuttgart
Bleckede	17 (74)							
Frankfurt	2	17 (74)						
Hamburg	0	0	18 (78)					
Hohne	2	1	3	13 (59)				
Landau	1	1	0	1	19 (86)			
Leonberg	2	2	2	5	0	13 (57)		
Lindau	1	0	1	0	1	1	17 (74)	
Stuttgart	1	2	1	0	0	1	2	17 (74)

The number of subpopulation-specific selective events was estimated by comparing one subpopulation for each of the eight subpopulations with the other seven subpopulation. For each stray subpopulation, the number (and percentage) of subpopulation- specific selective events are given.

## Discussion

Stray cats represent the largest cat population in the world and have extremely important genetic resources. Without human selection for aesthetic traits, stray cats exhibit a wide variety of phenotypes^28^. These cats have evolved under difficult conditions, having to resist numerous infectious and parasitic diseases and viruses in order to survive^29–31^. Therefore, high genetic variability and natural selection signatures of adaptability were expected in this population. The detection of ROHs allowed us to perform a comparative analysis of genomic diversity, inbreeding and selection signatures in non-breed and breed cat populations.

Here we present the first report on LD in a stray population from Germany. The stray population showed a rapid decay of LD. OSH and SIAM had a point of 50% decay of *r*^*2*^ with corresponding marker distances of 62.50 Kb (at *r*^*2*^ = 0.24) and 175.07 Kb (at *r*^*2*^ = 0.25), respectively. Stray showed a steeper decline in *r*^*2*^ values, reaching the 50% decay point at *r*^*2*^ = 0.23 for SNPs 1.12 Kb apart. Thus, compared to the results for cat breeds, stray belong to a more diverse population. In addition, we compared LD decay in stray with that in randomly bred populations from current and two other studies also using SNP array data^24,34^. Household in our study had a point of 50% *r*^*2*^ decay (at *r*^*2*^ = 0.25) at SNPs 4.55 Kb apart. Chinese wild cats and household cats from Germany had a point of 50% *r*^*2*^ decay at *r*^*2*^ = 0.24 with corresponding SNP distances of 36 kb and 19 kb, respectively^34^. The randomly bred population from the east and west reached their maximum *r*^*2*^ value 0.2 at the point of LD < 50 Kb. This population had a point of 50% *r*^*2*^ decay at *r*^*2*^ = 0.1 with a corresponding SNP distance of about 100 kb^24^. Compared to these randomly bred populations, the stray population had a faster decay of LD, indicating higher genetic diversity.

A small effective population size is a factor for high inbreeding coefficients. Due to the limited number of mating partners, the probability of mating with a related animal increases. The *N*_*e*_ of OSH (9) and SIAM (7) was lower than in many other cat breeds. In a study using the same DNA array with 13 breeds from Japan and the US, only SIAM from Japan and three other breeds from the US had a similar *N*_*e*_ (~ 10) as OSH and SIAM from Germany^35^. SIAM from the US had *N*_*e*_ above 100. Here we present the first report on *N*_*e*_ in stray. *N*_*e*_ of stray has dropped sharply from 1,923 to 55 in the last 100 generations, possibly due to the increasing prevalence of neutering surgery to render stray cats infertile. Neutering surgery can reduce the number of unfortunate cats. Considering that stray cats are crucial for future breeding under challenging conditions, as they represent an important genetic resource with various morphological, physiological and adaptive characteristics, we recommend preserving semen before neutering.

STR loci analyses showed average observed heterozygosities (*Ho*) from 0.49 to 0.74 in OSH populations^36–38^. The average *Ho* for OSH (0.722) based on the feline 63 K SNP array in this study falls into this range. In SIAM populations, the average SNP-based *Ho* was 0.20 using a panel of 148 evenly dispersed SNPs^39^, and the average STR-based *Ho* was from 0.47 to 0.64^36–39^. The average feline 63 K SNP array-based *Ho* for SIAM in this study indicated a higher value with *Ho* = 0.672 than the low-density SNP-/STR-based *Ho* results. This may imply that the feline 63 K SNP array may be more effective than low-density SNP sets/STRs. Compared to the studies of Australian household-stray cats (average *Ho* = 61% ± 5%)^27^, higher average heterozygosities were measured for stray cats (0.714) and household cats (0.704) from Germany. But this difference may be caused by different research strategies and methods.

The mean *F*_*IS*_ values in stray (0.052) and household (0.047) were positive. This could indicate inbreeding and an increase in homozygosity associated with natural selection of non-breed cats, albeit to a small extent. However, the *F*_*IS*_ values of stray individuals ranged widely range from -0.14 to 0.41, consistent with that of *F*_*ROH5*_, indicating that the population has a high genetic variability. It does not make much sense to look at the median or average value. The mean *F*_*IS*_ values < 0 in OSH (-0.031) and SIAM (-0.071) do not indicate a state of hybridization in the breeding populations, but may result from the low degree of variation of alleles in the whole genome caused by intensive artificial selection. In a study based on 10 STR loci, positive inbreeding coefficients were estimated for OSH (*F*_*IS*_ = 0.19) and SIAM (*F*_*IS*_ = 0.10)^37^. Of note is that these STR loci were selected for high polymorphism to be used in genetic individualization of domestic cat samples.

The genomic inbreeding coefficient *F*_*ROH*_ is indicative for inbreeding. There were significant differences between the *F*_*ROH5*_ in non-breed and breeding populations. Low *F*_*ROH5*_ values were found in stray (0.020 ± 0.022) and household (0.024 ± 0.024). This is in line with our expectations, because non-breed cats usually do not undergo artificial selection or breeding practices, but breed randomly in a large population. However, we found a few stray in the residential area of Frankfurt (n = 3), Hamburg (n = 1), Hohne (n = 1), Landau (n = 1) and Leonberg (n = 1) as well as one household with more than five ROH > 10 Mb and *F*_*ROH*_ > 0.07. Stray and household were randomly selected without prior knowledge where they came from, but without any similarity in their appearance with a breed cat. So, we can speculate whether recent inbreeding may had also occurred in stray and household and/or their ancestors were already inbred. As these individuals were seen in different residential areas, this may not be caused by selection of cats for this study. Very long ROH (> 25 Mb) may indicate recent inbreeding. Very few stray individuals with such long ROH were seen in the residential areas of Leonberg, Landau and Hohne. Distribution of cumulative genomic inbreeding coefficients across ROH lengths in stray indicated a steep increase to a value at 0.01 through short ROH segments (< 7.5 Mb) but a slow increase between values from 0.01 to 0.02 through longer ROH segments (> 7.5 Mb). This may suggest that approximately 50% of genomic inbreeding may be attributed to ancestral generations and in more recent generations increase of inbreeding steadily decreased in stray with very few exceptions of individual cats. A contrasting picture showed OSH and SIAM. In these breed cats, genomic inbreeding was caused through continued inbreeding up to recent generations. Breeding cats had significantly higher *F*_*ROH5*_, 0.069 ± 0.020 in OSH and 0.073 ± 0.015 in SIAM. These values were within the previously reported *F*_*ROH*_ range for OSH and SIAM (0.03–0.12)^11^. The low genetic diversity and high inbreeding coefficients in the breeding cat populations are probably the result of high selection pressure for certain aesthetic traits. We calculated the *F*_*ROH*_ across each of the three ROH length thresholds in SNPs, as the *F*_*ROH*_ takes on different values depending on the ROH length used for the calculation. Short ROHs capture ancient or historical inbreeding, and long ROHs reflect recent matings of related individuals as homozygous chromosomal segments are broken down over time by crossing-over in meiosis^40^. In stray and household, long ROH cover a similar proportion of the genome as short ROH, resulting in a similar *F*_*ROH*_ (*F*_*ROH60*_ ≈ *F*_*ROH30*_ ≈ *F*_*ROH5*_ ≈ 0.02 in stray and household, respectively). The long ROH make up the majority of ROH. The considerable amount of detectable long ROH in the OSH (*F*_*ROH5*_ = 0.07, *F*_*ROH60*_ = 0.05) and SIAM (*F*_*ROH5*_ = 0.07, *F*_*ROH60*_ = 0.06) breeds suggests that recent rather than distant inbreeding is likely to exist in breeding cat populations. It seems that the higher the inbreeding coefficient, the higher the consensus level of ROHs in the population (SIAM > OSH > household > stray). However, we could not find any ROH with 100% consensus in any of the four populations. It is conceivable that the few effective founders in each of the populations were distantly related and not yet inbred that much even in OSH and SIAM.

The *d*_*i*_ analysis identified nine stray unique selective regions in the genome where significant windows were only observed in stray, but not in household, OSH and SIAM. The region on chromosome B3 at position 114.9 Mb was identified as a signature of selection underlying feline biology and domestication by comparing domestic cat and wild cat *F*_*ST*_ results (*d*_*i*_)^23^. Next to this region, we detected a stray unique selective region on B3 at 115,036,645–115,982,934 bp using *d*_*i*_ analysis. It also overlapped with a partial consensus ROH shared by stray from eight different residential regions. The *GPHN* gene in this region encodes a neuronal assembly protein. Motor defects and hyperresponsiveness were observed in *GPHN*^–/–^ mice^41^. Hasan et al*.* reported that 12 of the 26 studied cat breeds showed a ROH island on chromosome B3 at 25–26 Mb^25^. In the same region, we found a signature of selection in stray using *d*_*i*_ analysis. The *GABRB3* gene in this region encodes the receptor for a major inhibitory neurotransmitter of the mammalian nervous system^42^. Considering the more aggressive and defensive behavior of stray cats, *GPHN and GABRB3* could be driving factors for the selection signature of stray in these regions. It was intriguing that the analyses in stray suggested high positive natural selection for survival and maintenance of non-breeds in harsh environment. The strongest candidate selective region overlapping 10% consensus ROH blocks is located on chromosome B3 (127 Mb) and contains four coding genes, i.e., *DIO2*, *CEP128*, *TSHR* and *GTF2A1*. The products of the *DIO2* and *TSHR* genes are essential for cold-induced thermogenesis in brown adipose tissue of small mammals^43,44^. GTF2A1 supports basal transcription of the *human T-cell leukemia virus type 1* (*HTLV-1*) promoter and is required for viral carcinogenesis^45,46^. Differential *TSHR* expression has been found during T-cell development in the thymus. Binding of thyrotropin to TSHR acts as a T-cell developmental factor in mice and humans^47^. Another strong candidate region on chromosome A1 (221 Mb) was also identified by the selective sweep and homozygosity mapping analyses. No coding gene is located in this region. Other candidate genes in other stray-specific selective regions, *PLCB2*, *BAHD1* and *TIGAR* were proposed to be involved in the host immune response. *PLCB2-/-* mice showed enhanced chemotaxis of various leukocyte populations and a sensitized in vivo response to bacteria, viruses, and immune complexes. PLCB2 plays a role in the response triggered by chemoattractants^48^. A bacterial protein targeting the BAHD1 chromatin complex can stimulate a type III interferon − mediated immune response to control bacterial colonization of the host. Murine listeriosis decreased in *BAHD1* + */–* mice^49^. TIGAR plays a dual role in cancer cell survival through regulating apoptosis and autophagy^50^. Furthermore, *BUB1B* and *FGF23* are involved in the regulation of lifespan, aging and reproduction. *BUB1B* is a central component of the mitotic spindle assembly checkpoint and protects against aneuploidy. Mouse models have revealed functions for *BUB1B* in the regulation of normal aging^51^. Its role in reproduction has been demonstrated by infertility in male and female *BUB1B* mutants^52^. *FGF23-/-* mice have a short lifespan and exhibit numerous biochemical and morphological features consistent with the premature aging-like phenotypes^53^. In summary, we propose that stray cats are subject to selection likely controlled by nature that affects adaptive thermogenesis, reproduction, immune system development, host immune responses to bacteria, viruses and chemoattractants, and lifespan and ageing. These biological processes are thought to play an important role in survival under harsh living conditions with bacteria, viruses, infectious and parasitic diseases in strays.

Twelve unique selective regions compared to SIAM have been identified in OSH by *d*_*i*_. The SIAM-derived OSH has a similar body shape and similar health problems to the modern SIAM, but has a wide range of coat colors and patterns. Consistent with this, five unique selective regions harboring pigmentation-related genes were discovered in OSH. One region on chromosome B4 at 70,056,541–72,975,983 bp falls into the candidate region under selection forming the coat color on Birman cats’ feet (11.25–114 Mb) identified by *d*_*i*_ estimates^5^. Of note, both the Birman and OSH breeds were derived from the Siamese, expanding its range of coat patterns and colors. *ADAMTS20* in this region was reported as a pigmentation candidate gene^54^. Mice homozygous for spontaneous or ENU-induced mutations in *ADAMTS20* exhibit abnormal coat or hair pigmentation, including a typical white belt phenotype^54^. Another candidate gene in this region, *KITLG*, encodes the ligand for KIT, which plays a central role in melanogenesis, melanoblast migration and proliferation. A regulatory region of the *KITLG* gene has been significantly associated with common blond hair color in Northern Europeans^55,56^. The largest OSH-specific selective region was on chromosome D1 at 42,138,904–50,969,224 bp and contained the coat color related gene *TYR*. The tyrosinase encoded by *TYR* catalyzes the first two steps and at least one subsequent step, in the conversion of tyrosine to melanin. Two mutations in *TYR* have been associated with the Siamese and Burmese coat color phenotypes^55^. Another gene related to color determination, *MITF*, was not found in the unique selective OSH regions. However, we identified *TYRO3* as a candidate gene linked to skin, as it has been reported to function as a *MITF* regulator in melanoma^57^. Other candidate genes located in the selective regions have been proposed to be involved in skin or eye pigmentation. *GPNMB* has been suggested to be important for melanin biosynthesis and development of the retinal pigment epithelium and iris^58^. Decreased expression of *FGF7* was found in pathological hypopigmentation of the skin^59^. Rab38cht*/cht* exhibited ocular hypopigmentation^60^. In addition, four genes *PDE4D*, *PKP2*, *NLGN1* and *ALDH1B1* may be candidate genes for congenital heart defects or gastrointestinal disorders in OSH. Three of these genes were not only located in unique selective OSH regions, but also in partially fixed ROH blocks. This could explain why congenital heart defects and gastrointestinal diseases are common in the OSH breed. Recent reports of *PDE4D* mutations suggest that functionality is an increased cardiac risk factor as well as increased risk of atrial fibrillation^61^. Mutations in *PKP2* have been reported to underlie familial arrhythmogenic right ventricular cardiomyopathy^62^. Down-regulated expression levels of neurexin and neuroligin in enteric nervous system may be involved in the pathogenesis of Hirschsprung disease^63^. ALDH1B1 has become an immunohistological marker for colorectal cancer^64^.

We have identified natural selection signatures in stray that are thought to influence adaptive thermogenesis, motor/neurological behavior, reproduction, immune system development, host immune response against bacteria, viruses and chemoattractant, as well as lifespan and aging. Artificial selection signatures in OSH have been found to likely be involved in pigmentation or cause the breed's common heart and gastrointestinal defects. Further research using whole genome sequencing data can foreseeably provide more definitive confirmations of the candidate selective regions presented. The candidate genes we have proposed also require an in-depth molecular investigation to reach satisfying conclusions.

## Conclusions

Compared to cat breeds, stray cats are a genetically diverse population with a rather low genomic inbreeding coefficient and a reduced increase of genomic inbreeding in recent generations. Ten genes have been identified in the unique selective regions of strays, when compared to household, OSH and SIAM, that are associated with reproduction, cold-induced thermogenesis, motor/neurological behavior, immune system development, viral carcinogenesis, host immune response against bacteria, viruses, chemoattractant and cancer cells, and lifespan and aging. Our results suggest, at least in part, selection for environmental adaptation and resistance to infectious disease in the stray population that is driven by nature rather than humans. In addition, eleven genes have been identified in the unique selective regions of the OSH, compared to the SIAM, that are implicated in the breed's wide range of coat colors and patterns, as well as its common heart and gastrointestinal defects. These selective regions and genes should be the subject of further investigation.

## Materials and methods

### Ethics declarations

In the present study, we used tissue samples from the biobank of our diagnostic lab. The living organisms were not involved in our study. Tissue samples from gonads were obtained from animal shelters where cats were neutered for other reasons. Oral swabs were sent to our diagnostic lab by cat breeders to get diagnostic test results. Therefore no animal welfare committee approval was obtained according to the German Animal Welfare Law (released on 05/18/2006, last changes on 03/29/2017).

### Samples and genotyping

Gonad samples of 150 stray and 26 household cats as well as oral swab samples of 21 unrelated SIAM and 49 unrelated OSH were obtained from the bio-bank of the Institute for Animal Breeding and Genetics at the University for Veterinary Medicine Hannover. These cats were random samples, mainly from Germany. The 49 OSH and 21 SIAM were not related with each other according to their pedigree records up to 4–6 generations. Genomic DNA was extracted using a standard ethanol fractionation with 6 M sodium chloride (NaCl) and 10% sodium dodecyl sulphate (SDS). The concentration of each DNA sample was adjusted to 50 ng/µL and then genotyped with the Illumina Infinium HD feline 63 K SNP BeadChip® Array (Illumina, San Diego, CA, USA) containing 62,897 SNPs.

### Statistical analysis

For the estimation of the genetic diversity measures, quality control was carried out using PLINK v.1.09 (www.cog-genomics.org/plink/1.9/)^65^. All SNPs from sex chromosomes and SNPs that could not be assigned to a chromosome were excluded from the dataset. Individuals with more than 30% missing genotype data were excluded from the analysis using *–mind* command in PLINK. *–maf* and *–geno* were set to their default values, i.e. 0.01 and 1 respectively. The final dataset contained 53,581 autosomal SNPs with a genotyping rate > 0.95 in 246 cats.

For each population independently, the following summary statistics were calculated using PLINK v.1.09 (www.cog-genomics.org/plink/1.9/)^65^, the function*–freq* was used to calculate (1) the number of monomorphic SNPs, and (2) the mean and standard deviation of minor allele frequency (MAF). The function*–hardy* was used to calculate (3) the mean and standard deviation of observed heterozygosity, and (4) the mean and standard deviation of expected heterozygosity. The number and frequency of all polymorphic SNPs for a dataset containing all individuals combined was determined using the PLINK function (*–freq*). The numbers of SNPs within different minor allele frequencies bins are reported.

Principal Components Analysis (PCA) of SIAM, OSH, stray and household cats was carried out in order to investigate population structure. The stray cats were found in eight regions across Germany (specifically, Bleckede, Frankfurt, Hamburg, Hohne, Landau, Leonberg, Lindau and Stuttgart). Using *–pca* command in PLINK, binary files were converted to eigenvec file and eigenval file. The eigenvec file was imported into R, v.4.1.0^66^ to generate PCA plots in three dimensions.

Using Net View package, v.3.4.1^67,68^ implemented in R, v.4.1.0^66^, a NetView analysis was performed to detect fine-scale stray population structure. It is based on a relationship matrix which was derived from SNP marker distances calculated with PLINK (www.cog-genomics.org/plink/1.9/), v.1.9^68,69^. NetView clusters based on mutual k-nearest neighbors were created to visualize the relatedness between the individuals^67,68^. The maximum number of nearest neighbors (K-NN) was analyzed to select suitable K-NN for the animal set. The visualization of the population network was done using Cytoscape, v.3.7.0^70^. To enable a visualization of potential connections between genetic distances and living regions, stray cats were grouped into 8 subpopulations according to their living regions (Bleckede, Frankfurt, Hamburg, Hohne, Landau, Leonberg, Lindau, and Stuttgart).

The effective population size *N*_*e*_ and the increase in inbreeding per generation *∆F* were calculated based on the linkage disequilibrium (LD) using PLINK v.1.09 (www.cog genomics.org/plink/1.9/)^65^. We computed the squared correlation (*r*^*2*^) as a measure of LD between SNP pairs per chromosome. The *r*^*2*^-values for SNP alleles with distances of 1 kb to 33.3 Mb were sorted into distance bins of 0.1 Mb. For every distance bin the mean *r*^*2*^-value was assessed. The *N*_*e*_ was calculated by ${N}_{e}= \frac{1-{r}^{2}}{4c{r}^{2}}$ , with c = recombination rate in Morgan units^71^. Regarding the distance c between two SNP markers, we assumed that 100 Mb was approximately 1 Morgan. The number of generations in the past (*t*) was assessed by $\frac{1}{2c}$ . *∆F* was estimated by $\Delta F= \frac{1}{2 {N}_{e}}$^72^.

Screening for ROH regions across the genome was conducted with the *–homozyg* command in PLINK v.1.09^65^, using a sliding window of ‘‘X’’ SNPs. A ROH in one individual was called if a homozygous stretch contained 5, 30, 60, or more SNPs, with the command *–homozyg-snp*. We did not allow for heterozygous SNPs and only admit five missing SNP genotypes per 60 SNP-window and three per 30 SNP window^73^. We determined ROHs that were partially fixed in each of the four populations and therefore common to 10%, 20%, 25%, 30%, 50%, 75% or 90% of all cats in a population. The genomic distribution of partial consensus ROHs in stray subpopulations was calculated using SAS, v.9.4 (Statistical Analysis System, Cary, NC, USA). Stray partial consensus ROHs were assigned into eight groups, i.e. Poolt*i*, a partial consensus ROH was shared by stray cats from *i* different living regions (*i* = 1, 2, 3, 4, 5, 6, 7, or 8). Percentage of each partial consensus ROH in a living region was estimated as ${n}_{r}/{n}_{t}$, with *n*_*t*_ = total number of stray cats from this region and *n*_*r*_ = number of stray cats harboring the ROH. A plot with ROH percentage values for all the eight regions on y-axis and the chromosomal positions on x-axis using ggplot2 package in R, v.4.1.0^66^ was plotted to represent the result.

The inbreeding coefficient *F*_*ROH*_ and fixation index *F*_*IS*_ were calculated using the software SAS, v.9.4 (Statistical Analysis System, Cary, NC, USA). *F*_*ROH*_ for each cat was estimated as a proportion of the length of all ROHs to the total length of all autosomes covered by SNPs: $FROH=\sum {L}_{ROH}/{L}_{AUTO}$^74^. *F*_*IS*_ for each individual ($i$) was estimated as:${F}_{IS,i}={O}_{i}-{E}_{i}/{nSNP}_{i}-{E}_{i}$ , with $Oi$ = number of observed homozygous SNPs, $Ei$ = number of expected homozygous SNPs and $n{SNP}_{i}$ = number of all autosomal SNPs genotyped in the individual cat^65^.

Locus specific divergence (*d*_*i*_) was previously developed and implemented based on unbiased estimates of pairwise *F*_*ST*_^75,76^. Original files (.ped and .map) were first converted into Variant Call Format (vcf) files using *–recode-vcf* command in Plink v1.9^69^. Pairwise *F*_*ST*_ was calculated using vcf files and *–weir-fst-pop* command of vcftools for each SNP between all populations. Subsequent calculations of *d*_*i*_ values were performed using scripts written in R using the following equation. For each SNP we calculated the statistic ${d}_{i}= \sum_{j\ne i}\frac{{F}_{ST}^{ij}-M({F}_{ST}^{ij})}{sd({F}_{ST}^{ij})}$, where $M({F}_{ST}^{ij})$ and $sd({F}_{ST}^{ij})$ denote the mean value and standard deviation of *F*_*ST*_ between populations (household vs OSH, household vs SIAM, OSH vs SIAM, stray vs household, stray vs OSH, and stray vs SIAM) from all the 53,581 SNPs. *d*_*i*_ was averaged over SNPs in nonoverlapping 1-Mb windows. Windows with less than four SNPs were discarded. The 99th percentile threshold was used to decide significance of the calculated *d*_*i*_ values. Using the same method, stray subpopulation specific selective events were identified by comparing the stray subpopulation to the remaining 7 stray subpopulations *F*_*ST*_ results (*d*_*i, stray_sub*_).

The selective region list and partially fixed ROH list were compared to search for overlaps present in both lists. We determined genes located in candidate regions using SAS, v.9.4 (Statistical Analysis System Institute Inc., Cary, NC, USA). PANTHER 15.0 (Protein Analysis Through Evolutionary Relationships)^77^ was utilized to analyze all genes located in the selective regions. The gene lists were investigated with the “functional classification” analysis and the “statistical overrepresentation” test. The overrepresentation test was performed on the annotation set “GO biological process complete”.

## Supplementary Information

Supplementary Information.

## Data Availability

The datasets analysed during the current study are available in the DRYAD repository, https://doi.org/10.5061/dryad.nvx0k6dwd (https://datadryad.org).

## Abbreviations

Stray: Stray population
household: Household population
OSH: OSH population
SIAM: SIAM population
DIO2: Iodothyronine Deiodinase 2
*CEP128*: Centrosomal protein 128
*TSHR*: Thyroid stimulating hormone receptor
*GTF2A1*: General transcription factor IIA Subunit 1
*PLCB2*: Phospholipase C Beta 2
*BAHD1*: Bromo adjacent homology domain containing 1
*TIGAR*: TP53 induced glycolysis regulatory phosphatase
*BUB1B*: BUB1 mitotic checkpoint serine/threonine kinase B
*FGF23*: Fibroblast growth factor 23
*ADAMTS20*: ADAM metallopeptidase with thrombospondin type 1 motif 20
*KITLG*: KIT ligand
*KIT*: KIT proto-oncogene, receptor tyrosine kinase
*TYR*: Tyrosinase
*MITF*: Melanocyte inducing transcription factor
*TYRO3*: TYRO3 protein tyrosine kinase
*GPNMB*: Glycoprotein Nmb
*FGF7*: Fibroblast growth factor 7
*RAB38*: RAB38-member RAS oncogene family
*PDE4D*: Phosphodiesterase4D
*PKP2*: Plakophilin-2
*NLGN1*: Neuroligin 1
*ALDH1B1*: Aldehyde dehydrogenase 1B1
