# Supplementary material for: Tracking footprints of artificial and natural selection signatures in breeding and non-breeding cats
Source: Sci Rep. 2022 Oct 27;12:18061. doi: 10.1038/s41598-022-22155-7 (PMC9613910; doi:10.1038/s41598-022-22155-7)
Supplement: Supplementary file 1 — Supplementary Information. [file 41598_2022_22155_MOESM1_ESM.zip › 28082022_Supplementary information.docx]

**Supplementary Materials:**


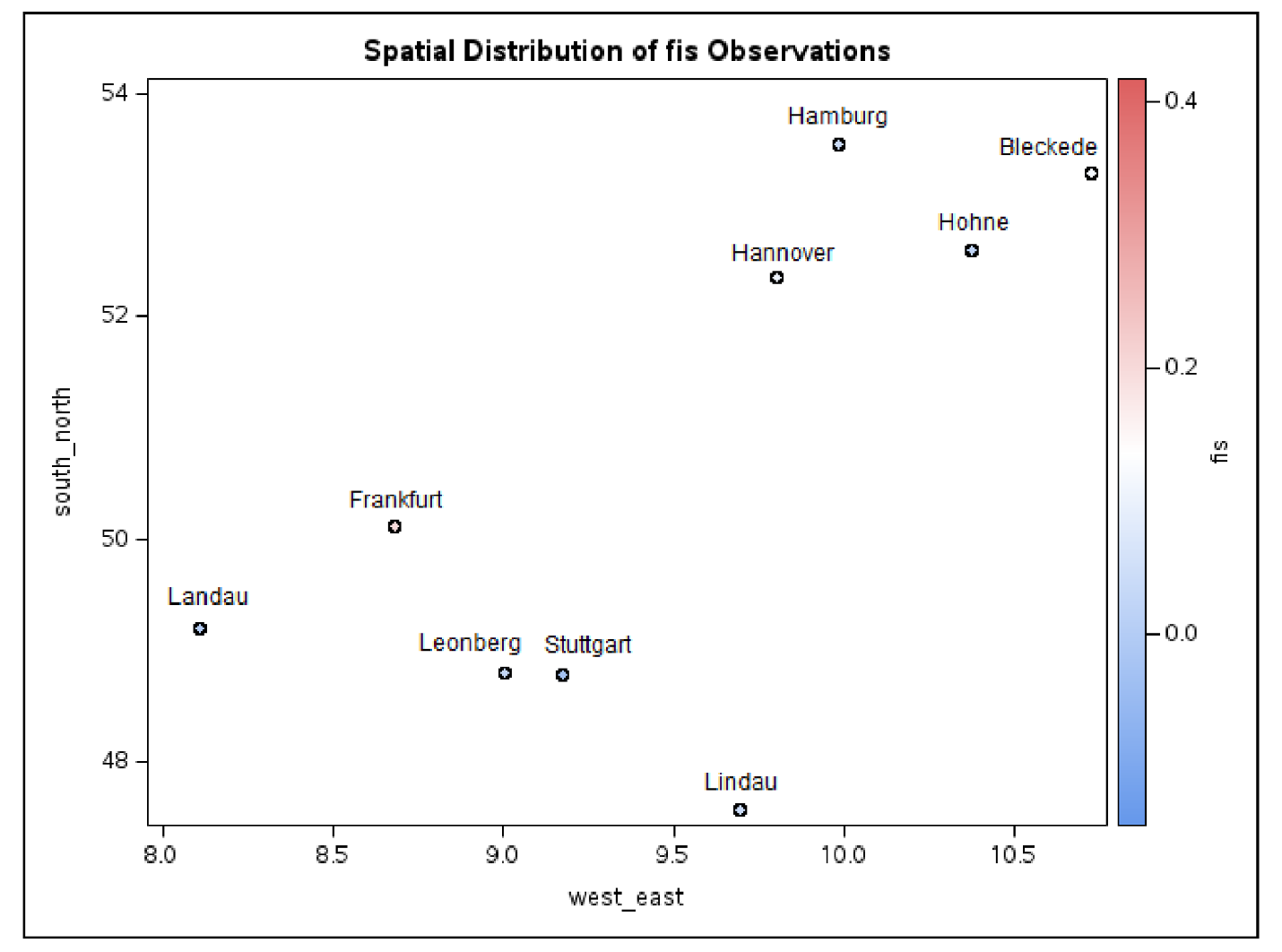


**Figure S1** Spatial distributions of *F_IS_* by stray subpopulations (Bleckede, Frankfurt, Hamburg, Hohne, Landau, Leonberg, Lindau and Stuttgart) and household (Hannover) are presented according to the coordinates of their living regions. They are represented by colored circles according to their *F_IS_* values.

**
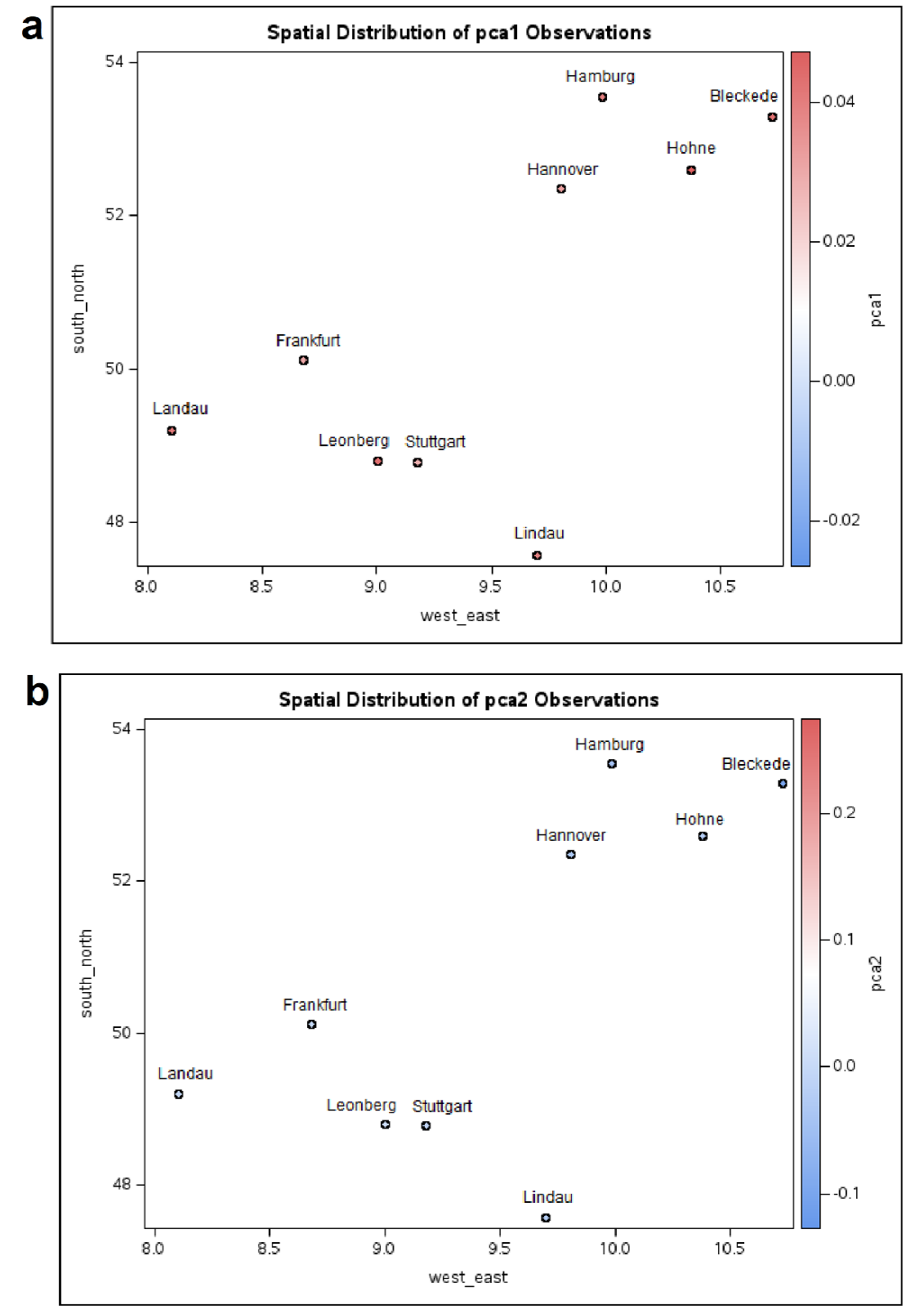
**

**Figure S2** Spatial distributions of PC1 (**a**) and PC2 (**b**) by stray subpopulations (Bleckede, Frankfurt, Hamburg, Hohne, Landau, Leonberg, Lindau and Stuttgart) and household (Hannover) are presented according to their coordinates of their living regions. They are represented by colored circles according to their PC values

**
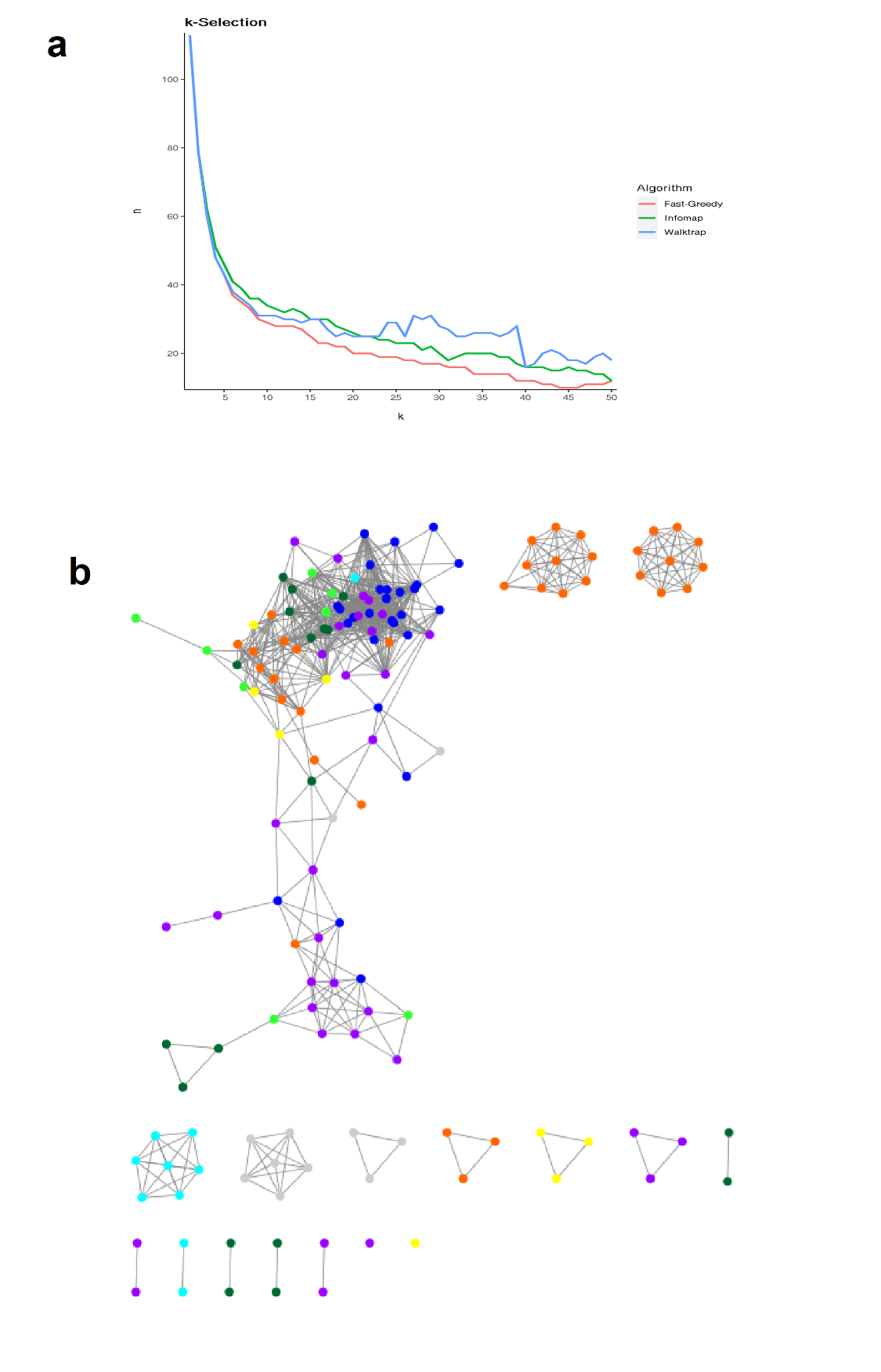
**

**Figure S3** Visualization of the fine-scale population network for stray subpopulations. (**a**) K selection. (**b**) Netview figure with K-NN = 25, grey: Bleckede, purple: Frankfurt, dark blue: Hohne, dark green: Landau, light green: Leonberg, orange: Lindau, yellow: Stuttgart, light blue: Hamburg.


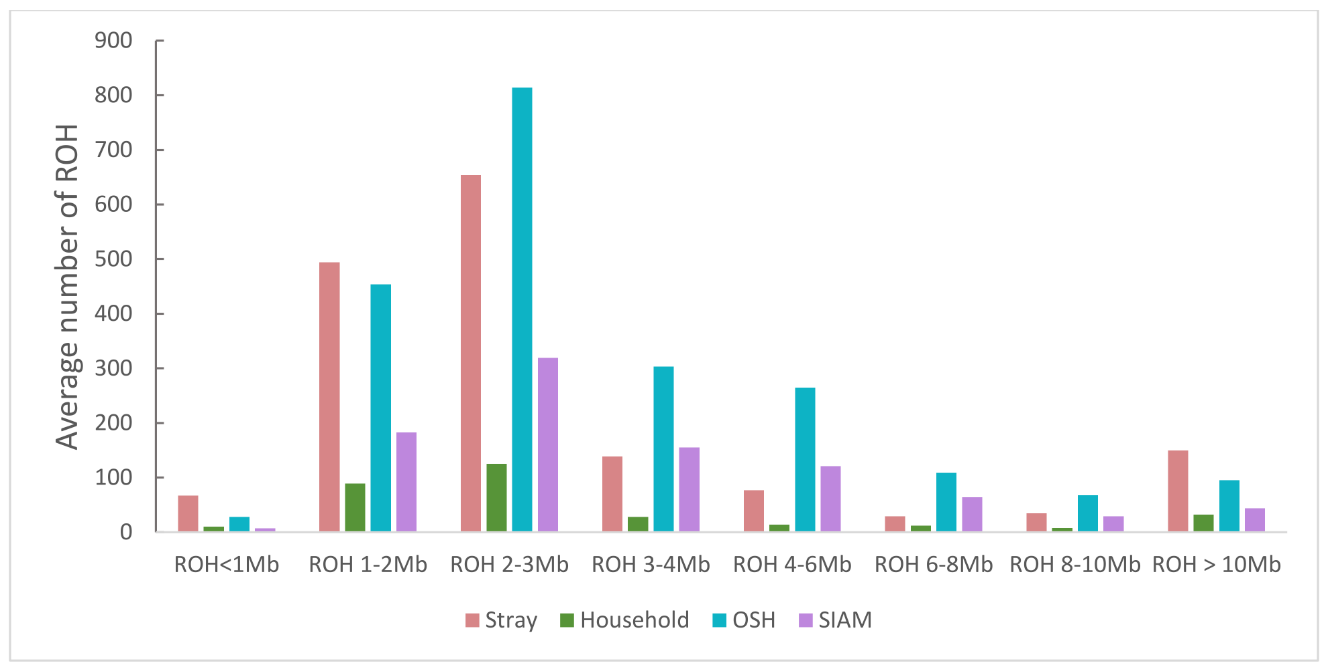


**Figure S4** Average number of runs of homozygosity (ROHs) detected over *ROH5* in eight different length ranges in individuals of each population (stray, household, OSH and SIAM).

**
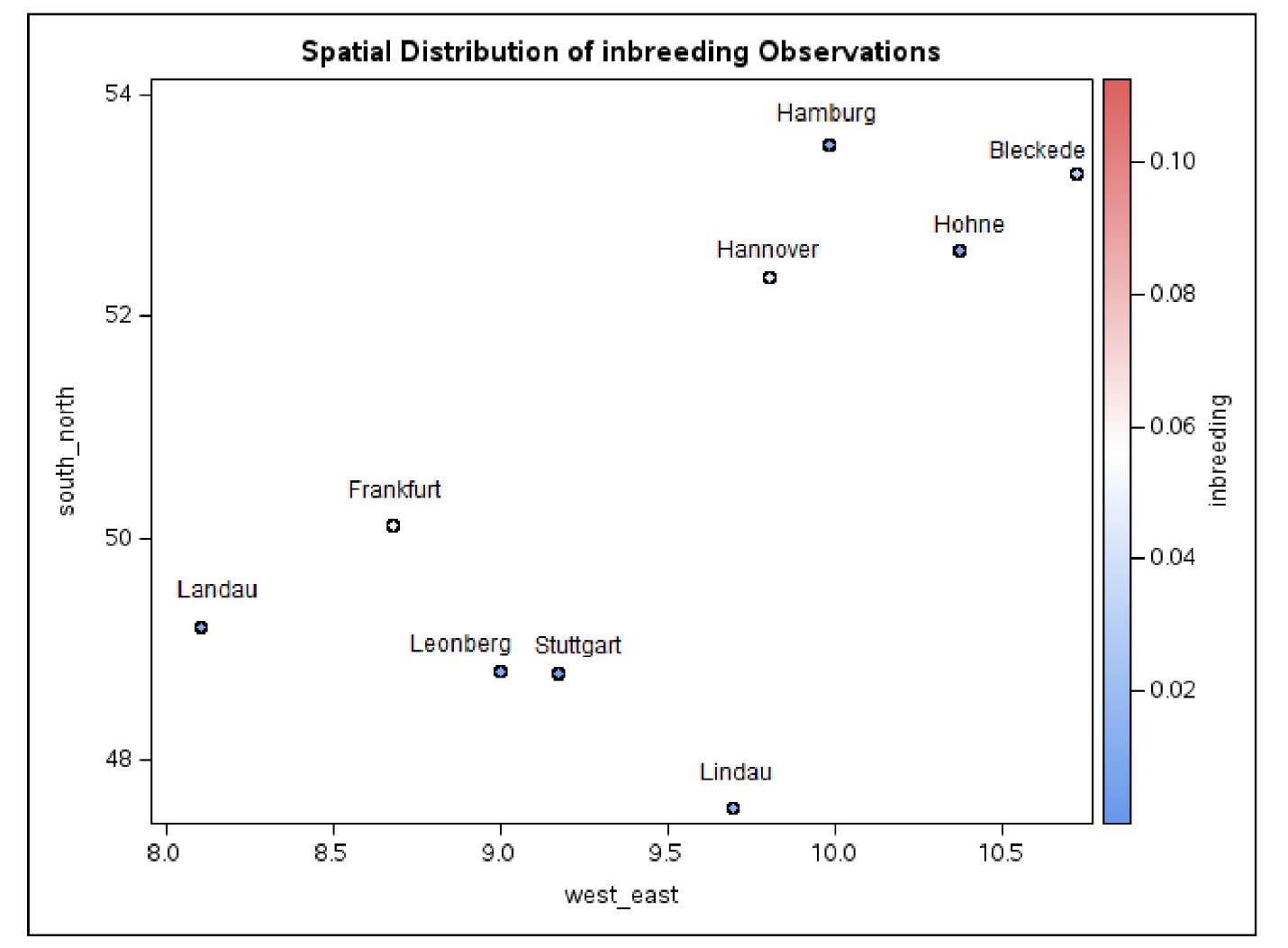
**

**Figure S5** Spatial distributions of genomic inbreeding coefficients calculated from *F_ROH5_* for stray subpopulations (Bleckede, Frankfurt, Hamburg, Hohne, Landau, Leonberg, Lindau and Stuttgart) and household (Hannover) are presented according to the coordinates of their living regions. They are represented by colored circles according to their *F_ROH5_* values.


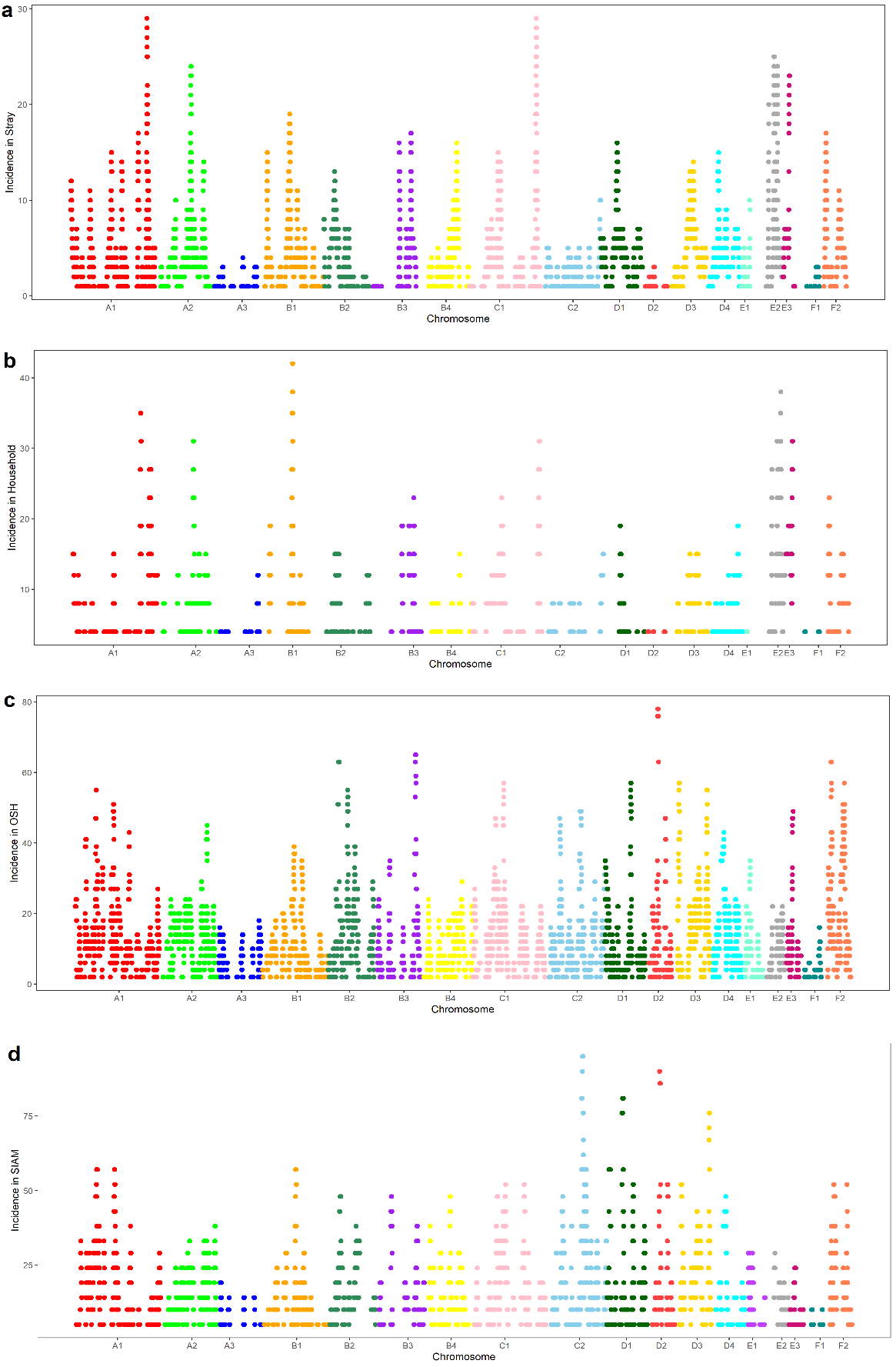


**Figure S6** Incidence-plots of the single nucleotide polymorphisms (SNP) from ROH5 per chromosome for stray (**a**), household (**b**), OSH (**c**) and SIAM (**d**), respectively. Given is the incidence based on the position of each SNP.

**a**
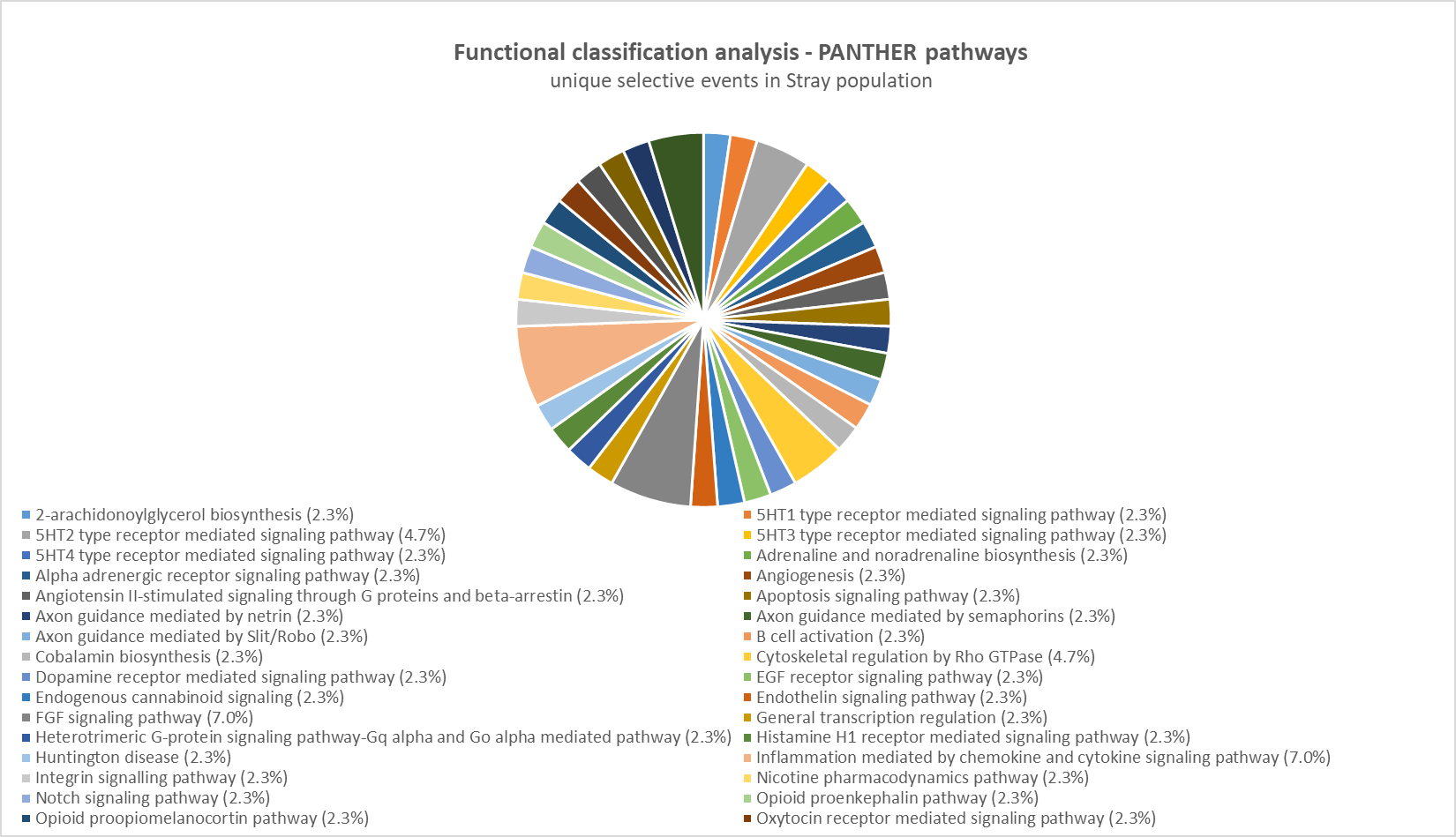


**b**
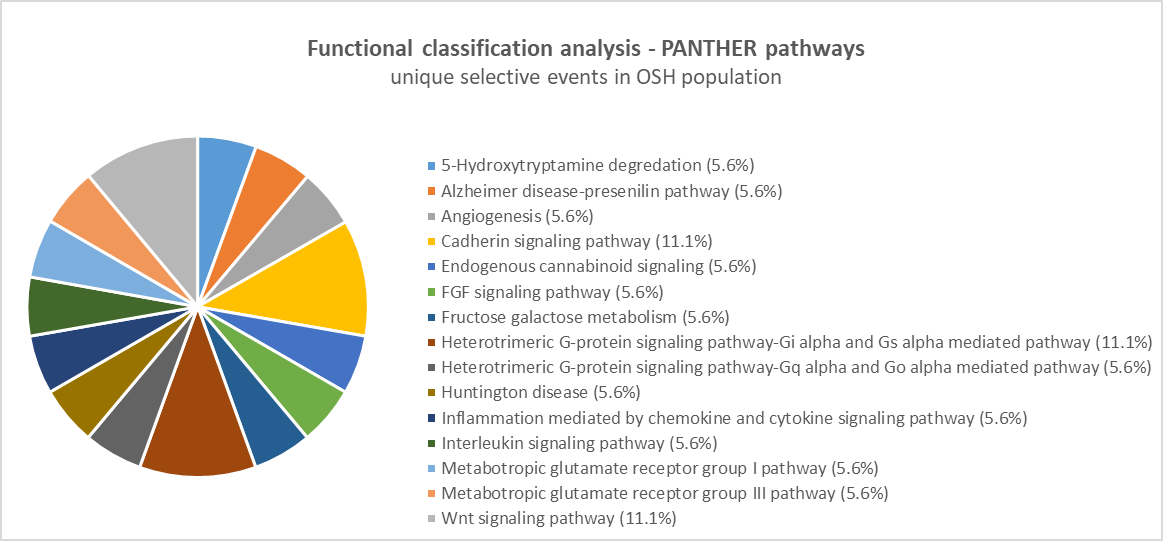


**Figure S7** Results of the PANTHER functional classification analysis for the genes located in unique selective events in the stray (**a**) and OSH (**b**) population, respectively. Listed are the pathways.

**Table S1** Overview of candidate regions under selection in cat populations.

| Candidate region | Population | Phenotype | Ref. |
| --- | --- | --- | --- |
| A1:117 Mb | domestic cats (compared to wild congeners) | Multiple | [1] |
| A1:193 Mb |  |  |  |
| B3: 33 Mb |  |  |  |
| B3: 114.9 Mb |  |  |  |
| D3:73 Mb |  |  |  |
| B3:25-29 Mb | British Shorthair, Burmese, Munchkin, Norwegian Forest Cat, Oriental, Peterbald, Scottish Fold, Selkirk Rex, Siamese, Siberian, Tenessee Rex | Multiple | [2] |
| E2:37-38 Mb | Bengal, Colony, LaPerm, Lykoi, Norwegian Forest Cat, Scottish Fold, Selkirk Rex, |  |  |
| D4:31-33 Mb | LaPerm, Munchkin, Turkish Van |  |  |
| A2: 41,154,844–42,509,338 | Persian | Brachycephaly | [3] |
| A2:44,595,242–45,638,786 |  |  |  |
| C2:127,112,224–128,268,366 |  |  |  |
| A2: 101.75-103 Mb | Persian | Brachycephaly | [4] |
| C1: 163.25-164.75 Mb |  |  |  |
| A1: 188.25-189.25 Mb | Maine Coon | Eating behavior/size |  |
| D2: 79.5-81.25 Mb | Bengal | Coat type |  |
| E3: 43.75-44.75 Mb | Turkish Van | Van color |  |
| E3: 41.25-44.75 Mb | American Curl | Curled ears |  |
| F1: 63.5-45 Mb |  |  |  |
| B1: 147.25-151 Mb | La Perm | Curly coat |  |
| A1: 82-89 Mb | Ragdoll | Multiple |  |
| B4: 11.25-114 Mb | Birman | Gloves |  |
| D3: 1.25-2.25 Mb | Peterbald, Oriental and Siamese | Slender body |  |

**Table S2** Overview of results and methods for demography studies of random breeding cat populations.

| Random bred populations | *n* | LD at 50%  Decay | | MAF | | Inbreeding | | | *Ne* | | BeadChip | Ref. |
| --- | --- | --- | --- | --- | --- | --- | --- | --- | --- | --- | --- | --- |
|  |  | *r^2^* | Distance (Kb) | |  | *F_ROH_* | *F_ST_* | *F_IS_* | Contemp. | Past |  |  |
| Chinese feral cats | 22 | 0.24 | 36 | ~13% 0<MAF<0.05 | |  |  |  |  |  | custom illumina array consisting of 1536 SNPs | [5] |
| Household cats from  Hamburg, Germany | 21 | 0.24 | 19 | ~9% 0<MAF<0.05 | |  |  |  |  |  | custom illumina array consisting of 1536 SNPs | [5] |
| Random bred population from east and west | 270 | 0.1 | ~100 | 0.22 | |  |  | 0.096 |  |  | Infinium Feline 63 K iSelect DNA array | [6] |
| Random bred population from Japan | 110 |  |  | 0.19 | | ~0.54 | ~0.24 |  | ~600 | Contemp. Ne > past Ne | Infinium Feline 63 K iSelect DNA array | [7] |
| Random bred population from USA | 212 |  |  | 0.21 | | ~0.51 | ~0.24 |  | ~300 | Contemp. Ne < past Ne | Infinium Feline 63 K iSelect DNA array | [7] |
| Feral cats  from Australia | 126±3 |  |  |  | |  |  | 0.09±0.05 |  |  | 15 STR genomic markers | [8] |
| House/stray cats from  Australia | 75±2 |  |  |  | |  |  | 0.14±0.05 |  |  | 15 STR genomic markers | [8] |
| Random bred cats over  African | 10 |  |  |  | |  |  | 0.30 |  |  | 38 microsatellites | [9] |
| Arabian wildcats | 5 |  |  |  | |  |  | 0.11 |  |  | 38 microsatellites | [9] |
| Random bred cats over  Europe | 10 |  |  |  | |  |  | 0.47 |  |  | 38 microsatellites | [9] |

LD, linkage disequilibrium; *r^2^*, squared correlation coefficient; MAF, minor allele frequency; *F_ROH_*, inbreeding estimates based on runs of homozygosity; *F_IS_*, inbreeding coefficient of an individual relative to the subpopulation; *F_ST_*, measure of population differentiation due to genetic structure; *Ne*, estimates of the effective population size; Contemp., contemporary; Past, 20 generations ago; STR, short tandem repeat

**Table S3** *F_IS_* values by stray subpopulations from Bleckede, Frankfurt, Hamburg, Hohne, Landau, Leonberg, Lindau and Stuttgart.

**Table S4** The number of ROHs per chromosome for each population (stray, household, OSH and SIAM).

**Table S5-S8** Genes located within ROHs of different degree of consensus in each population (stray, household, OSH and SIAM). Given are the cat chromosome considered, the start and end position of the ROH on this chromosome, the estimated ROH length, the number of SNPs and all genes located in the ROHs.

**Table S9** Partial consensus ROHs identified in the stray population. Given are the cat chromosome considered, the start and end position of the ROH on this chromosome, the number of animals containing the ROHs, and the distribution of animals (number and percentage) in different residential regions.

**Table S10** *F_ROH5_* comparisons among stray subpopulations from Bleckede, Frankfurt, Hamburg, Hohne, Landau, Leonberg, Lindau and Stuttgart.

**Table S11** PANTHER statistical overrepresentation test. Genes located in unique selective events in stray, or OSH, respectively, were analyzed with the overrepresentation analysis tool of PANTHER 15.0. Given are the biological processes the enriched genes involved in, their fold enrichment and the false discovery rate (*p*-value).

**Table S12** Stray subpopulation-specific selective events identified for each of the eight stray subpopulations. Given are the cat chromosome considered, the start and end position of the selective events on this chromosome, and the genes located in the selective events.

# References

1. Montague, M. J.*, et al.*, Comparative analysis of the domestic cat genome reveals genetic signatures underlying feline biology and domestication*.* *Proc Natl Acad Sci U S A*. **111**, 17230-17235 (2014).

2. Gorssen, W., R. Meyermans, S. Janssens, and N. Buys, A publicly available repository of ROH islands reveals signatures of selection in different livestock and pet species*.* *Genet Sel Evol*. **53**, 2 (2021).

3. Bertolini, F.*, et al.*, Evidence of selection signatures that shape the Persian cat breed*.* *Mamm Genome*. **27**, 156-157 (2016).

4. Alhaddad, H., M. Abdi, and L. A. Lyons, Patterns of allele frequency differences among domestic cat breeds assessed by a 63K SNP array*.* *Plos One*. **16**, (2021).

5. Alhaddad, H.*, et al.*, Extent of linkage disequilibrium in the domestic cat, Felis silvestris catus, and its breeds*.* *PLoS One*. **8**, e53537 (2013).

6. Gandolfi, B.*, et al.*, Applications and efficiencies of the first cat 63K DNA array*.* *Sci Rep*. **8**, 7024 (2018).

7. Matsumoto, Y.*, et al.*, Genetic relationships and inbreeding levels among geographically distant populations of Felis catus from Japan and the United States*.* *Genomics*. **113**, 104-110 (2021).

8. Spencer, P. B. S.*, et al.*, The Population Origins and Expansion of Feral Cats in Australia*.* *J Hered*. **107**, 104-114 (2015).

9. Lipinski, M. J.*, et al.*, The ascent of cat breeds: genetic evaluations of breeds and worldwide random-bred populations*.* *Genomics*. **91**, 12-21 (2008).
